# Validation of Reference Genes for Studying Different Abiotic Stresses in Oat (*Avena sativa* L.) by RT-qPCR

**DOI:** 10.3390/plants10071272

**Published:** 2021-06-22

**Authors:** Judit Tajti, Magda Pál, Tibor Janda

**Affiliations:** Department of Plant Physiology, Agricultural Institute, Centre for Agricultural Research, ELKH, H-2462 Martonvásár, Hungary; pal.magda@atk.hu (M.P.); janda.tibor@atk.hu (T.J.)

**Keywords:** reference gene validation, RT-qPCR, abiotic stress, oat

## Abstract

Oat (*Avena sativa* L.) is a widely cultivated cereal with high nutritional value and it is grown mainly in temperate regions. The number of studies dealing with gene expression changes in oat continues to increase, and to obtain reliable RT-qPCR results it is essential to establish and use reference genes with the least possible influence caused by experimental conditions. However, no detailed study has been conducted on reference genes in different tissues of oat under diverse abiotic stress conditions. In our work, nine candidate reference genes (ACT, TUB, CYP, GAPD, UBC, EF1, TBP, ADPR, PGD) were chosen and analysed by four statistical methods (GeNorm, Normfinder, BestKeeper, RefFinder). Samples were taken from two tissues (leaves and roots) of 13-day-old oat plants exposed to five abiotic stresses (drought, salt, heavy metal, low and high temperatures). ADPR was the top-rated reference gene for all samples, while different genes proved to be the most stable depending on tissue type and treatment combinations. TUB and EF1 were most affected by the treatments in general. Validation of reference genes was carried out by PAL expression analysis, which further confirmed their reliability. These results can contribute to reliable gene expression studies for future research in cultivated oat.

## 1. Introduction

Oat (*Avena sativa* L.) is cultivated throughout the world as a unique cereal which serves as an excellent forage due to its high protein and essential mineral level [1]. It is also a good source of dietary fiber, especially β-glucan, with the potential to improve human health [2]. However, this crop is less profitable than maize, soybean, or wheat crops, so it is often cultivated in areas which have a number of disadvantages, like drought or high salinity [3]. Furthermore, this species is sensitive to changes in light and temperature as well [4]. The current understanding of the stress-adaptive mechanisms in oat on a molecular level is still limited, and the main reason for that is probably that, first and foremost, the genomes of the most important crops were sequenced, like rice [5,6] maize [7], and wheat [8], which further helped to properly investigate these species. Nowadays, more and more attention is being paid to oat because of its economic benefits. The fully sequenced and assembled oat genome only became available recently (in 2020), which will revolutionize oat-related research. The discovery of genes playing role in abiotic stress response of oat is still ahead of us and the examination of the related gene expression changes is a great way to better understanding gene functions in oat under adverse environmental conditions.

Although microarrays and RNAseq represent a preferred choice when it comes to the investigation of gene function on a wider scale [9], quantitative real-time PCR (RT-qPCR) is still of great importance when the methods presented above need further validation, or if only a small number of genes has to be analysed, because of its cost-effectiveness and relative simplicity. RT-qPCR uses cDNA as a template, which is created by the reverse transcription of mRNA, and different fluorescent dyes, which allow to correlate PCR product concentration to fluorescent intensity. Cq (quantification cycle) value means the number of cycles, where the florescence intensity exceeds the background fluorescence. Thus, lower Cq value means higher PCR product concentration. The quantification of the obtained results can be done in two ways. The expression level of the target genes can be calculated by using an absolute quantification where serially diluted standards with known concentrations are used to create a standard curve. Thus, the concentration of the unknown samples can be determined based on their Cq values. The other possible way, as in our work, is the relative quantification, where the changes of the target gene expression are normalized by one or more internal control genes, also known as reference genes [10]. RT-qPCR is a powerful and sensitive method, however, it is only the case, when the experimental settings are properly executed and appropriate normalization methods are used [11]. In addition to the sample integrity and the specificity of the reaction, the stability of the applied reference gene greatly determines the reliability of the measurements [12,13]. Therefore, it is crucial to identify such reference genes that are stably expressed, and whose expressions are least affected by experimental conditions. Several modern tools have been developed to find the best reference genes, such as GeNorm [14], Normfinder [15], BestKeeper [12], dCt method [13], or RefFinder [16], and each one of them uses different calculations.

In the last few years, several studies were conducted to investigate the reference gene stability in oat under various circumstances. Jarošová and Kundu [17] examined the stability of different reference gene candidates in virus-infected wheat, barley and oat. Furthermore, stable reference genes under herbicide stress were identified in wild relatives of cultivated oat [18,19], while the effects of different tissues and developmental stages were also examined [19,20]. Reference gene stability under salt stress was investigated by Duan et al. [21]. However, no detailed study was conducted on the effects of various abiotic stresses on the stability of reference gene candidates. In our study, the expression stabilities of nine generally used candidate reference genes, namely ACT (Actin), TUB (α-Tubulin), CYP (Cyclophylin), GAPDH (Glyceraldehyde 3-phosphate dehydrogenase), UBC (Ubiquitin conjugating enzyme), EF1 (Elongation factor 1-α), TBPII (TATA-binding protein II subunit), ADPR (ADP-ribosylation factor), and PGD (Phosphogluconate dehydrogenase), were tested in the leaves and roots of two oat cultivars (winter and spring varieties) under drought, salt, heavy metal, cold, and heat treatments. Four different statistical methods (GeNorm, Normfinder, BestKeeper, RefFinder) were used to identify the most stable reference genes. For the final validation of candidate reference genes, PAL (Phenylalanine ammonia lyase) was chosen as target gene, which encodes a highly stress responsive enzyme playing key role in the biosynthesis of a major stress hormone, salicylic acid (SA). Our results provide a basis for the normalization of gene expression in oat.

## 2. Results

### 2.1. Verification of Amplification Products, Primer Specificity and Amplification Specificity

**Figure 1:**
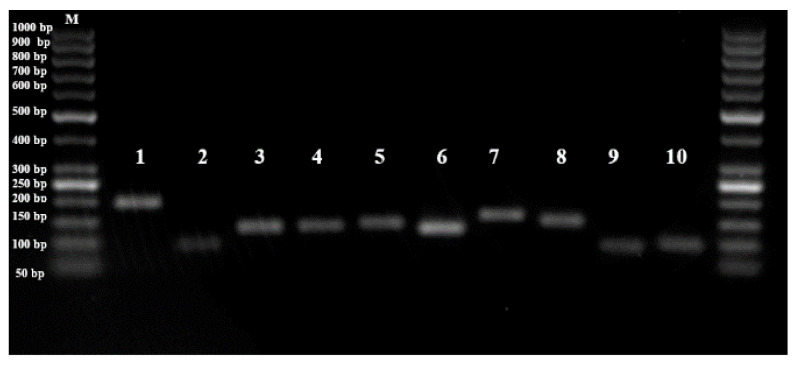
PCR products of reference genes (1–9) and gene of interest (10). M: 250 bp DNA Ladder, 1: ACT, 2: TUB, 3: CYP, 4: GAPDH, 5: UBC, 6: EF1, 7: TBPII, 8: ADPR, 9: PGD, 10: PAL.

Amplicon sizes of nine reference genes (ACT, TUB, CYP, GAPDH, UBC, EF1, TBPII, ADPR, PGD, PAL) and target gene (PAL) were checked by running 5-5 µL of PCR products on 1.5% agarose gel. All PCR products were clearly amplified with the expected amplicon sizes, with no impurities or primer dimers (Figure 1).

**Figure 2:**
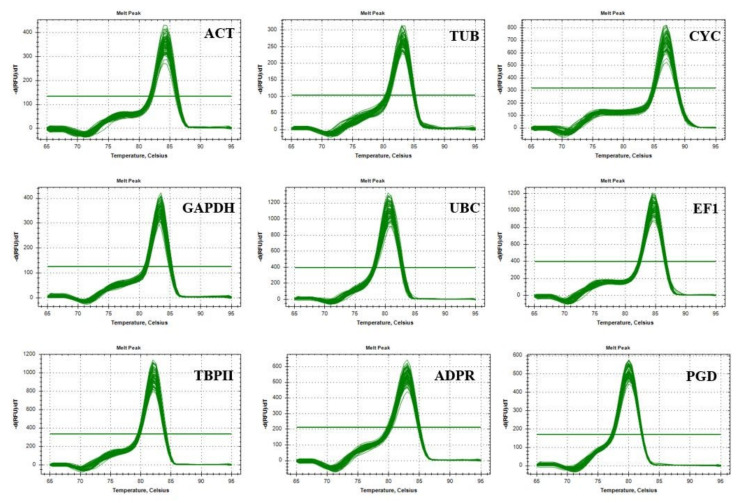
Melt curves of RT-qPCR amplification of nine candidate reference genes (ACT, TUB, CYC, GAPDH, UBC, EF1, TBPII, ADPR, PGD) in leaves and roots of Mv Pehely and Mv Hópehely under different abiotic stresses.

Melt curve analysis of the nine reference genes revealed that in every case a single peak was observable under different abiotic stresses in both oat genotypes, and the amplification curves showed good repeatability (Figure 2), which means that the primers amplified a single PCR product. Therefore, they are appropriate for detailed RT-qPCR studies.

**Figure 3:**
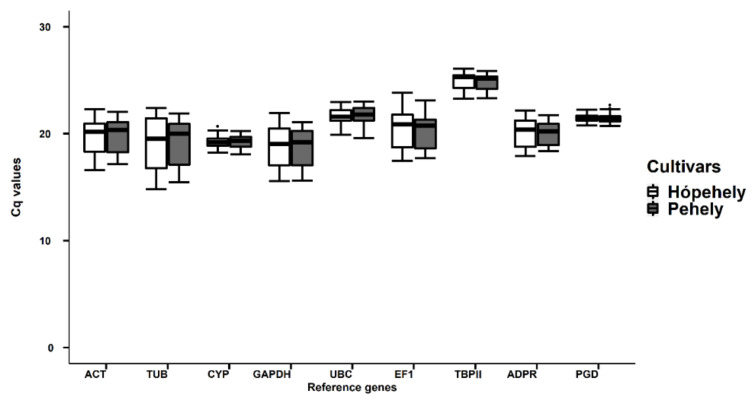
qRT-PCR Cq values for nine oat candidate reference genes in leaf and root samples under different abiotic stresses in the investigated cultivars. White boxplots present the Cq values of cultivar Mv Hópehely, while grey boxplots show the Cq values of Mv Pehely.

Cq values of the nine reference genes in all oat samples are shown in the boxplot (Figure 3). Comparing the Cq value ranges for the two investigated cultivars shows they are very similar for a certain reference gene. The range of Cq values in all samples varied from 14.83 to 26.54, which shows that the mRNA transcript levels of the reference genes were high enough for gene expression analysis (<35). However, Cq values were influenced by treatments, tissue type, and in some cases by genotype, so it was necessary to analyse them under different circumstances.

### 2.2. Evaluation of Stability Ranking for Candidate Reference Genes

#### 2.2.1. GeNorm Analysis

The M values were calculated by GeNorm to determinate the average expression stability of the nine reference genes in Mv Pehely (Table 1) and Mv Hópehely (Table 2). A reference gene with an M value under the threshold 1.5 can be considered as stable. In both genotypes ADPR had relatively high stability in most treatments and tissue types, while EF1 and TUB usually located at the end of the ranking order. For drought stress, ACT and ADPR were the most stable in leaves, while in roots ADPR and GAPDH ranked the highest in terms of stability. Interestingly, UBC had the lowest M values in Mv Hópehely, but it was less stable in Mv Pehely. PGD and CYP were among the most stable genes in salt stressed leaves in both genotypes, while in roots UBC and ADPR had the lowest M values. Under Cd stress ACT and ADPR had the highest stability values in leaves, while GAPDH was the most stable in roots. For cold stress, ADPR was at the beginning of ranking order in both cultivars in all tissue types. ACT was the most stable in the leaves under cold in Mv Pehely. However, it had lower stability in Mv Hópehely. UBC was among the first three most stable genes in the roots of both genotypes under cold stress. In the leaves of heat stressed samples, ACT and ADPR had the lowest M values in hydroponic cultures, but in pot experiment CYP proved to be the second most stable in both cultivars. In the roots, ADPR and EF1 had the highest stabilities in both genotypes under heat stress.

**Figure 4:**
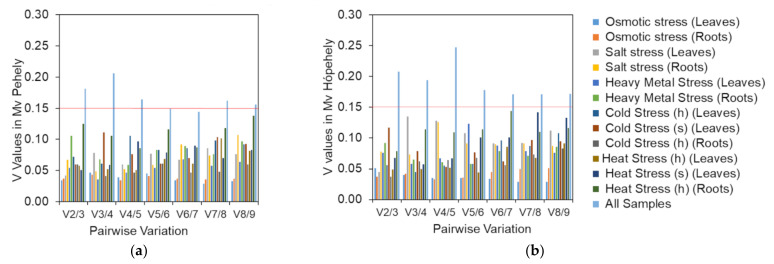
Pairwise variation measurement of candidate reference genes in the two oat cultivars: (**a**) V values in Mv Pehely; (**b**) V values in Mv Hópehely. The two types of growing media are indicated with h (hydroponic) and s (soil).

GeNorm can be used to determine the optimal number of references genes needed for normalisation by pairwise variation measurement (V_n_/V_n+1_). A V_n_/V_n+1_ with 0.15 cutoff value indicates that the addition of and extra reference gene is not necessary. In our study, the V_2_/V_3_ values in both genotypes were lower than 0.15 in both tissue types under different abiotic stresses (Figure 4), with the exception of all treatment/tissue combinations, which means that two genes are enough for normalisation when dealing with a certain abiotic stress. If all the samples need to be analysed together, five reference genes are required for optimal normalisation for Mv Pehely, because only the V_5_/V_6_ value was lower than 0.15. However, for Mv Hópehely the V_8_/V_9_ value was still higher than 0.15, indicating that all the nine reference genes could be necessary for normalisation under various stress conditions in this genotype. In conclusion, it may worth choosing the most appropriate reference genes according to the applied experimental conditions.

#### 2.2.2. NormFinder Analysis

Normfinder evaluates the stability value by determining inter- and intragroup variations and the lower stability value indicates a more stable reference gene. Stability values are presented in Table 3 (Mv Pehely) and Table 4 (Mv Hópehely). When drought stress was applied, both the genotype and tissue type had an effect on the expression stability. In drought-stressed leaves of Mv Pehely PGD was the most stable, while it was ranked only as fourth in the leaves of Mv Hópehely. GAPDH was less stable in the leaves of both investigated cultivars, while in roots it was on the second place in Mv Pehely, while on the first place in Mv Hópehely in terms of ranking order. However, under drought stress, all the stability values were very low, which indicates that the treatment only slightly influenced the stability in general. Under salt stress, GADPH was the most stable in leaves, while PGD and ADPR had the highest stability in roots. In Cd-stressed leaves, GAPDH and ADPR had the top ranking, while in the roots, the genotype influenced the stability of the investigated genes as, in Mv Pehely EF1 was the most stable, while in Mv Hópehely, GAPDH had the lowest stability values. For temperature stresses, ADPR was the most stable reference gene candidate in both cultivars.

#### 2.2.3. Bestkeeper Analysis

Bestkeeper analyses the expression of the candidate reference genes by the calculation of the standard deviation (SD) and the coefficient of variance (CV) using the untransformed Cq values. The reference gene with the lowest CV ± SD value can be considered the most stable. CV ± SD values are presented in Table 5 for Mv Pehely and in Table 6 for Mv Hópehely. Bestkeeper ranked in most cases PGD as the most stable reference gene, while TUB was mostly at the end of the ranking order. In drought-stressed samples PGD was ranked in the first three places in leaves and in roots as well. For salt stress, TBPII could be considered as a stable reference gene. Under Cd stress, PGD was in the first three places of the ranking order in both genotypes and organs. However, the stability of UBC highly depended on the tissue type. It was very stable in leaves while unstable in roots. TBPII could be considered as a stable gene in Cd-stressed leaves while CYP in roots in both genotypes. During temperature stresses, PGD and TBPII were amongst the top-rated genes.

#### 2.2.4. RefFinder Analysis

RefFinder is a user-friendly online tool, which combines the so far presented statistical methods (geNorm, Normfinder, BestKeeper, and the dCt method) in order to calculate a final comprehensive ranking. Ranking orders according to stress treatments in different tissues are shown in Table 7 for Mv Pehely and in Table 8 for Mv Hópehely. ADPR proved to be the most stable and TUB the most unstable in most treatments in both tissue types in general, while the stability of the other reference genes was influenced to a greater extent by the applied treatment, tissue, and genotype combinations. In drought-stressed leaves of Mv Pehely, PGD was the most stable, while in Hópehely, ACT was the top-ranked, and in roots, GAPDH and ADPR were the most stable genes. When applying salt stress, in leaves GADPH and CYP were the most stable, while in roots ADPR and PGD were at the beginning of the ranking order. For heavy metal stress, GAPDH and ADPR were the most stable genes in leaves, but PGD and CYP in roots. Under cold and heat stresses, ADPR was almost always the top-ranked one in both genotypes and tissue types. Furthermore, ADPR proved to be stable under different growing conditions as well when the plants were exposed to temperature stresses.

### 2.3. Validation of the Reference Gene Candidates

**Figure 5:**
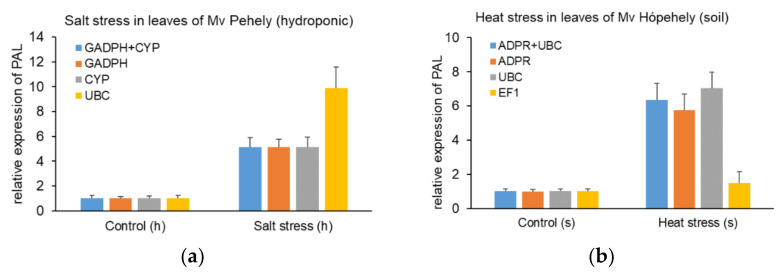
Relative expression levels of the target gene. (**a**) Relative expression level of phenylalanine ammonia lyase (PAL) under heat stress in leaves of Mv Hópehely in pot experiment using the two most stable (ADPR and UBC) and the least stable (TUB) genes. (**b**) Relative expression level of PAL under salt stress in leaves of Mv Pehely in hydroponic culture using the two most stable (GAPDH + CYP) and the least stable (UBC) genes.

The relative expression level of PAL (phenylalanine ammonia lyase) was used to validate the candidate reference genes in our study. PAL is one of the key enzymes during the synthesis of the well-known stress hormone, SA [22]. Elevated PAL activity or mRNA level was found in different plant species after exposure to various abiotic stresses [23,24,25]. The relative expression level of PAL was normalized with the two most stable reference genes and the least stable reference gene in oat according to the applied stress factor. Reference genes were carefully chosen after comparing the results of different evaluation programs while taking into account the effects of genotype and tissue type as well. As shown in Figure 5A, in response to heat in the leaves of Mv Hópehely growing in soil, the relative expression of PAL increased significantly, when using the most stable reference genes (ADPR + UBC), while the relative transcript level did not reach a two-fold increase when using the least stable reference gene, TUB. When applying salt stress in the leaves of Mv Pehely hydroponically (Figure 5B), a five-fold increase was measured when applying the most stable reference genes alone or in combination (GAPDH + CYP), but the relative expression level was overestimated, when UBC was used as a reference gene. In conclusion, if the applied reference gene has very high stability, one gene is sufficient for normalisation. The relative expression values of PAL in all tested tissues and genotypes under different abiotic stresses are available in Supplementary Figures S1–S4.

## 3. Discussion

RT-qPCR is a widely used method for gene expression analysis because of its relative simplicity and high sensitivity. However, the reliability of the results is greatly determined by reference gene selection used for normalization [11,14]. Ideally, the stability of the reference gene should not be influenced by the experimental conditions. Nevertheless, several studies point out that the mRNA transcript levels could be affected by tissue type [19,20], developmental stage [26], treatment type [27,28], and genotype [26] as well. Furthermore, the expression stability of the same reference gene may also depend on the investigated species [17]. Therefore, it is always necessary to analyse and validate the potential reference genes prior to their applications.

In our study, nine candidate reference genes (ACT, TUB, CYP, GAPD, UBC, EF1, TBP, ADPR, PGD) were screened from the leaves and roots of two oat genotypes under different abiotic stresses (drought, salt, heavy metal, cold and heat), and their expression stabilities were analysed by four different statistical programs (GeNorm, NormFinder, Bestkeeper, RefFinder). The general rating by the different programs had a substantial agreement, which were the least stable genes for each treatment/tissue/genotype combinations so they could be easily excluded. However, the most stable genes determined by the applied programs were not always the same due to their different calculations. It was especially remarkable when using BestKeeper, which gave a higher ranking to a certain gene compared to the other programs in some cases. That was especially true for TBPII in drought-stressed roots of Mv Hópehely, and for PGD in cold stressed leaves of both cultivars, respectively. The difference between the ranking order of Bestkeeper and the rest of the softwares is also mentioned by other studies [29,30,31]. The stability of the reference genes were influenced by all the experimental conditions, such as tissue type, genotype, and treatment type, but the applied stress treatments had the most pronounced effect on stability in general.

For drought stress, all the stability values were very low, independently from the applied program which indicates that gene expression stabilities of the reference genes was not particularly affected by this treatment in general. The suitability of ADPR under drought stress was confirmed by our experiments and it was also mentioned earlier in barley (*Hordeum vulgare*, L.) [32]. Furthermore, CYP was found to be stable in barley when exposed to drought, while in our experiment it was one of the least stable genes. Interestingly, a study in durum wheat *(Triticum durum*, L.) identified GAPDH as a stable reference gene under drought [33], although under our experimental conditions it only showed high stability in roots, while it was unstable in the leaves of the investigated oat cultivars. In addition to our study, the applicability of ACT as a reference gene in drought-stressed leaves was also suggested in barley [32]. Interestingly, the stability values of UBC were genotype and tissue type dependent. Namely, it was the least stable gene in the leaves of Mv Pehely, while it was one of most stable ones in the leaves of Mv Hópehely. Furthermore, it had moderate stability in the roots of Mv Pehely while it was stably expressed in the roots of Mv Hópehely.

When applying salt stress, tissue type greatly affected the expression stability. GAPDH, CYP and PGD had high stability in leaves, while in roots UBC and ADPR were the most stably expressed genes in both cultivars. GAPDH was a proposed reference gene in *Triticum durum* [33] under salt stress as well. However, the effects of salt stress were also examined in oat by Duan et al. [21], who found the expression of GAPDH unstable. In contrast, the same study identified EF1 and TBP as the best combination for normalization, but in our experimental setup both genes had relatively low stabilities. ACT exhibited low expression stability in oat both in our study and in the above mentioned work [21]. Furthermore, α-TUB was also at the end of ranking order in our analysis and in the above-mentioned study as well. The expression stability of UBC showed genotype and tissue type dependence under salt stress similarly to drought stress, which is also an osmotic stress.

Under Cd stress, GAPDH was found to be stably expressed according to the different evaluating softwares in our study. However, in giant reed (*Arundo donax* L.), which also belongs to the *Poaceae* family, GAPDH was amongst the least stable genes when the plants were treated with Cd [34]. ACT1 showed high stability in leaves of both investigated oat cultivars. In agreement with this, other members of the Actin gene family showed high expression stabilities under Cd exposure, ACT12 in switchgrass (*Panicum virgatum* L.) [31] and ACT3 in soybean (*Glycine max* L.) [35]. PGD was a stable reference gene in the roots of both oat genotypes, however, in soybean its expression was greatly influenced by Cd stress [35]. TUB exhibited low expression stability in our oat samples and in Cd-treated soybean plants [35] as well.

When looking for stably expressed genes under temperature stresses, two oat homologs of wheat ADPR (Ta2291) and PGD (Ta30797) were also tested, since they were suggested previously as suitable reference genes candidates [27]. The overall applicability of ADPR for cold and heat stress in different genotypes, tissue types, and growing media was confirmed since it was at the first three places of the ranking order calculated by every software, with the exception of Bestkeeper. The high expression stability of ADPR under cold and heat stress was also indicated in *Hordeum brevisubulatum* [36] and it was the best reference gene in cold stressed barley [32]. However, PGD only exhibited high expression stability in the roots of Mv Pehely under cold stress, while it was less stable in leaves and under heat stress. The stability of ACT was influenced by genotype, tissue type, and growing medium. Specifically, it showed the high stability in the leaves when cold stress was applied in Mv Pehely, and under heat stress, but only when the plants were grown hydroponically. ACT is a commonly used reference gene in different plant species [29], but these differences suggest a cautious approach when choosing this gene as a reference gene. EF1 was usually amongst the least stable genes under temperature stresses, with the exception of the heat stressed roots of Mv Pehely where it was the most stable gene. EF1 was also ranked the highest in terms of stability in *Hordeum brevisubulatum* under heat stress [36].

In order to validate the stability of the investigated reference genes, PAL expression analysis was performed. Our study revealed, as long as a reference gene with very high stability is chosen (Figure 5), one reference gene may be sufficient for accurate normalization. However, according to the GeNorm V/V value calculation the application of two reference genes are more suitable in leaves or in roots exposed to certain abiotic stress. As shown in Supplementary Figures S1–S4, the expression of PAL was highly inducible by most of the treatments, especially in leaves. However, drought stress might be considered as a mild stress in this study, which is supported by the fact that PAL expression was not induced by PEG treatment nor in leaves or roots of the two genotypes. Correspondingly, the reference gene stability values calculated by the different software were very low in the case of all reference genes tested compared to the values under other abiotic stresses. In contrast, salt treatment, heavy metal exposure, and low temperature caused a remarkable elevation of PAL transcript levels in both genotypes, which indicates an increased stress effect on the plants. Accordingly, the stability of the reference gene candidates changed to a higher extent depending on the individual genes. Interestingly, heat stress only induced PAL expression in the leaves of Mv Hópehely when grown in soil but not in hydroponic culture. However, this treatment greatly affected reference gene stability in general. In agreement with this observation, the growing media influenced the stability of certain reference genes as well. While ADPR kept its high stability under both growing conditions, as indicated by the different software, the ranking order of the rest of the reference gene candidates changed according to the growing medium. For example, ACT showed high stability in the leaves of heat-stressed Mv Pehely in hydroponic culture, however in soil it was less stable. Furthermore, when investigating the stability in the cold-stressed leaves of Mv Hópehely, CYP was only stable in soil, but its expression was influenced by cold temperature under hydroponic growing conditions.

## 4. Conclusions

For the first time, our work aimed to identify stable reference genes for gene expression studies under different abiotic stresses in cultivated oat. Since many studies mention that genotype can also greatly influence the stability of a certain reference gene, a winter and a spring oat cultivar were chosen with different expected sensitivities to abiotic stresses, at least in terms of temperature stresses. Nine candidate reference genes were tested in two different tissues (leaves and roots) under drought, salt, heavy metal, cold, and heat exposures. In order to compare the stabilities of the candidate reference genes, four different algorithms were used, and their results were compared to find the best candidate according to treatment and tissue type. Although in most cases there was agreement on the ranking order of the used programs, Bestkeeper proposed different reference gene as most stable on several occasions compared to the other applied tools. Furthermore, genotype effect was clearly observable on reference gene stability, with the exception of the most stable ones which are discussed below. Our results confirmed previous findings in that the stability of certain reference genes could be greatly influenced by the different experimental conditions. Moreover, the possible effect of growing media under temperature stresses on reference gene stability was also revealed.

For all samples, ADPR was the top-ranked gene in both cultivars. For individual stress treatments, the most stable reference genes varied according to the tissue type and genotype. However, efforts were made to propose the most appropriate reference gene candidates according to the treatment type. Under drought stress, ADPR was a suitable reference gene in leaves, while GADPH in roots. For salt stress, GAPDH and PGD were appropriate reference genes in leaves, while ADPR in roots. When the plants were exposed to heavy metal stress, GAPDH was stably expressed in leaves and PDG had high stability in roots. ADPR performed the best in cold and heat-stressed samples. Nevertheless, α-TUB and EF1-α were influenced greatly by the experimental conditions and they were rarely suitable for normalization. In addition, our study further confirmed that the application of other traditionally used housekeeping genes, such as ACT or CYC, should be carefully considered, since their expressions were unstable in most cases. For the first time, stably expressed reference genes for different tissues were identified in a spring and winter oat genotype under five different abiotic stresses. Our study provides a suitable reference for selecting stable internal reference gene candidates to investigate gene expression under various abiotic stresses in oat. These results can contribute to better understanding the molecular mechanisms playing a role in abiotic stress response of this species.

## 5. Materials and Methods

### 5.1. Plant Materials, Growth Conditions, and Stress Treatments

Two oat (*Avena sativa* L.) cvs. ‘Mv Pehely’ (spring type) and ‘Mv Hópehely’ (winter type) were selected for the experiments. Seeds were germinated for three days on wet filter under dark conditions at 25 °C. Thereafter, they were grown either hydroponically using modified Hoagland solution [37] or in plastic pots filled with a 3:1 (*v:v*) mixture of loamy soil and sand at 22 °C/20 °C with 16-h/8-h light/dark photoperiod, 250 µmol m^−2^ s^−1^ light intensity and 75% relative humidity in a Conviron PGV-36 phytochamber (Controlled Environments, Winnipeg, Canada). The above-mentioned parameters are referred to as control conditions. For hydroponic experiments glass beakers were used with 10 plants per beaker, for pot experiment also 10 plants were sown in one pot. Nutrient solution for hydroponic culture was changed in every second day, while for the plants growing in pot regular water supply was provided. The 13-day-old plants were exposed to different abiotic stresses for 24 h. Stress conditions were chosen based on earlier findings, where stress responses were clearly observable, but they were not very serious causing the death of the plants. For drought stress, plants were treated with 15% PEG-6000 hydroponically [38] while salinity stress was induced by adding 250 mM/L NaCl [39] to the hydroponic medium. For heavy metal treatment plants were treated with modified Hoagland solution containing 250 µM/L Cd(NO_3_)_2_ [40]. In order to determine if different growing conditions can cause any change in the stability of reference genes when applying temperature stresses, hydroponically grown and soil-grown plants were also tested. Cold stress was imposed at 4 °C while heat stress was applied at 35 °C in growing chambers where all settings were the same as control conditions, with the exception of temperature. Meanwhile, one part of the hydroponic cultures were treated with Hoagland solution (hydroponic control) and were kept together with untreated plants of the soil experiment (soil control) under control conditions for 24 h. For leaf samples the second, fully developed ones were collected while root tissues were sampled only from hydroponically grown plants after washing the roots with distilled water. Thereafter, all samples were frozen immediately in liquid nitrogen and stored at −80 °C until further analyses.

### 5.2. Plant Materials, Growth Conditions, and Stress Treatments Isolation of RNA and cDNA Synthesis

Total RNA was extracted from leaf and root samples with TRI Reagent. Samples were further cleaned with Direct-Zol RNA MiniPrep Kit (Zymo Research, Irvine, CA, USA) including on-column Dnase I treatment. RNA quantification was carried out with a Nanodrop 2000 Spectrophotometer (Thermo Fisher Scientific Inc., Wilmington, DE, USA) while its integrity and the lack of gDNA contamination was checked on 1.5% agarose gel. Then, 1000 ng of total RNA was reversely transcribed into cDNA with M-MLV Reverse Transcriptase (Promega Corporation, Madison, WI, USA) and oligo (dT)18 (Thermo Fisher Scientific Inc., Wilmington, MA, USA) according to the manufacturer’s instructions. Four-fold dilution products were stored at −20 °C until RT-qPCR studies.

### 5.3. Reference Gene Selection and PCR Primer Design

The majority of candidate reference genes were chosen based on existing literature that utilised them as suitable stable internal control genes for qRT-PCR analysis in different plant species [18,21,28,41]. The applicability of ADPR (Unigene cluster: Ta30797) and PGD (Unigene cluster: Ta2291) (candidate reference genes proposed by Paolacci et al. [27]) were confirmed previously by our research group in wheat (*Triticum aestivum* L.) under different stress conditions [40,42,43,44]. For primer design, known wheat sequences were chosen either from NCBI Gene Bank or in the case of ADPR and PGD the corresponding sequences were obtained from NCBI-Unigene database. Since the full reference genome of the hexaploid oat was released first-ever only in late 2020 ([45] it was not yet available at the time of our primer design, homologous oat sequences were retrieved from the Avena sativa v1.0 genome database (https://avenagenome.org, BioProject 541449, accessed on 21 June 2021) via BLASTn search using highly conserved regions (identification of conserved domains was performed via NCBI Conserved Domain Finder [46]) of the corresponding *Triticum aestivum* cDNA sequence with the following criteria: aligned sequence size being >300 bp in length, an E-value < 1 × 10^−5^ and minimum 87% sequence homology to the query sequence (detailed information about sequence alignments, E-values, and percentage of identity is available in Supplementary Figures S5–S14).

For primer design Primer3 software [47] was used and primers were also checked with Oligoanalyzer [48] to avoid primer dimerization. The primer design conditions were as follows: Tm, 59–62 °C; amplicon size, 90–200 bp; primer length, 20–24 bp; GC content, 45–60%. PCR products were also run on 1.5% agarose gel in order to confirm the presence of a single amplicon with the expected amplicon size. Primer sequences are available in Table 9.

### 5.4. Real Time Quantitative PCR and Amplification Efficiency Determination

Measurements were performed on a Biorad CFX96 Touch Real-Time Detection System in 96-well microtiter plates. Mastermix was prepared using a final volume of 5 µL, which consisted of 1 µL 4-fold diluted cDNA, 200 nM forward and reverse primers, 2.5 µL PCRBIO SyGreen Mix (PCR Biosystems Ltd., London, United Kingdom) and 2.5 µL molecular grade water. PCR cycling consisted of three steps as follows: 3 min initial pre-incubation at 95 °C, followed by 39 cycles of 5 s at 95 °C for denaturation, and 30 s at 60 °C for annealing and extension. Melting curve analysis was also performed to verify PCR specificity by constant increase in temperature from 65 °C to 95 °C, at increment of 0.5 °C. A 5-step dilution series of cDNA pool (including cDNA samples from different genotypes and tissue types exposed to 5 abiotic stresses) was used for standard curve preparation. PCR efficiency and correlation coefficient (R^2^) were determined for each gene by CFX Maestro program. All reactions were run using three biological and three technical replicates. No template controls were also included to check the absence of primer dimers and random contaminations.

### 5.5. Stability Ranking of Reference Genes

To evaluate the relative expression stabilities of candidate reference genes under different abiotic stresses, four different statistical softwares, GeNorm [14], Normfinder [15], BestKeeper [12] and an online data analysis tool, RefFinder [16] were used. Cq values obtained from CFX Maestro program were exported into Microsoft Excel 2016 and used as input for further analyses. The GeNorm algorithm was applied as a built-in module of qBasePLUS software to evaluate the stability of reference genes based on stability value (M). Genes with an M value below the threshold of 1.5 are considered as stably expressed. The software can also determine the optimal number of reference genes for target gene expression normalisation with pairwise variation calculation (V_n_/V_n+1_). A V_n_/V_n+1_ cutoff value of 0.15 or lower means that the addition of a further reference gene is not necessary. NormFinder was used as an Excel-based algorithm to identify stable reference genes based on intra- and inter-group variations amongst the tested genes and the lowest stability value indicates the highest stability. The program needs the raw Ct values to be transformed using the formula 2^−∆Cq^. Bestkeeper is also an Excel-based tool which uses raw Cq values as input. It can rank the stability values by calculating the coefficient of variation (CV) and standard deviation (CV ± SD). Reference genes with the lowest CV ± SD values can be considered as the most stable ones. A comprehensive ranking of the above-mentioned evaluating programs was prepared with RefFinder which assigns an appropriate weight to an individual gene and calculates the geometric mean of their weights for the overall final ranking.

### 5.6. Validation of Reference Genes by Expression Analysis of PAL under Different Abiotic Stresses

In order to validate the reliability and stability of the candidate reference genes determined by the applied software, PAL was selected as target gene to analyse the gene expression pattern using the two most stable reference genes and the least stable reference gene. Relative transcript level was calculated with the 2^−ΔΔCt^ method [49]. The expression level of PAL was determined with forward primer 5′-GCAACTTCCAGGGCACCC-3′ and reverse primer 5′-CTCCGAGAACTGAGCGAACAT-3′ (reference sequence MT150275.1, amplicon size 95 bp, amplification efficiency 93%).

## Figures and Tables

**Table 1:** Average expression stability values (M) of nine reference gene candidates calculated by GeNorm in Mv Pehely. The two types of growing media are indicated with h (hydroponic) and s (soil), while h. is the abbreviation of heavy.

Rank	Osmotic Stress		Salt Stress		H. Metal Stress		Cold Stress			Heat Stress			All Samples
	Leaves (h)	Roots (h)	Leaves (h)	Roots (h)	Leaves (h)	Roots (h)	Leaves (h)	Leaves (s)	Roots (h)	Leaves (h)	Leaves (s)	Roots (h)	
1	ADPR	GAPD	GAPD	UBC	ACT	GAPD	ACT	ACT	GAPD	ACT	TUB	ADPR	TBPII
	0.101	0.079	0.106	0.193	0.126	0.182	0.175	0.151	0.145	0.188	0.145	0.286	0.503
2	ACT	ADPR	CYP	ADPR	CYP	ACT	ADPR	ADPR	ADPR	ADPR	CYP	EF1	UBC
	0.102	0.081	0.110	0.195	0.133	0.199	0.181	0.159	0.150	0.189	0.156	0.298	0.533
3	TBPII	ACT	PGD	CYP	ADPR	CYP	UBC	CYP	UBC	GAPD	ACT	PGD	ADPR
	0.106	0.092	0.117	0.203	0.145	0.239	0.196	0.166	0.163	0.192	0.159	0.328	0.552
4	CYP	UBC	ACT	TBPII	GAPD	PGD	CYP	TBPII	PGD	UBC	EF1	ACT	ACT
	0.150	0.134	0.219	0.215	0.154	0.269	0.230	0.309	0.175	0.209	0.204	0.391	0.706
5	PGD	PGD	ADPR	EF1	PGD	EF1	TUB	TUB	TBPII	CYP	ADPR	TUB	EF1
	0.177	0.156	0.253	0.244	0.192	0.298	0.355	0.352	0.206	0.236	0.319	0.428	0.785
6	GAPD	TBPII	EF1	PGD	EF1	ADPR	GAPD	PGD	ACT	PGD	UBC	CYP	GAPD
	0.212	0.188	0.325	0.290	0.241	0.373	0.415	0.371	0.264	0.300	0.373	0.524	0.861
7	EF1	CYP	TUB	GAPD	TBPII	TBPII	EF1	UBC	EF1	TBPII	TBPII	TBPII	TUB
	0.228	0.216	0.370	0.396	0.311	0.450	0.476	0.410	0.294	0.340	0.448	0.563	0.934
8	TUB	TUB	TBPII	ACT	UBC	UBC	PGD	GAPD	TUB	EF1	GAPD	UBC	CYP
	0.236	0.242	0.452	0.449	0.360	0.497	0.565	0.517	0.322	0.460	0.481	0.664	1.048
9	UBC	EF1	UBC	TUB	TUB	TUB	TBPII	EF1	CYP	TUB	PGD	GAPD	PGD
	0.254	0.271	0.507	0.565	0.413	0.583	0.632	0.591	0.378	0.528	0.544	0.795	1.143

**Table 2:** Average expression stability values (M) of nine reference gene candidates calculated by GeNorm in Mv Hópehely. The two types of growing media are indicated with h (hydroponic) and s (soil), while h. is the abbreviation of heavy.

Rank	Osmotic Stress		Salt Stress		H. Metal Stress		Cold Stress			Heat Stress			All Samples
	Leaves (h)	Roots (h)	Leaves (h)	Roots (h)	Leaves (h)	Roots (h)	Leaves (h)	Leaves (s)	Roots (h)	Leaves (h)	Leaves (s)	Roots (h)	
1	UBC	UBC	PGD	UBC	ADPR	GAPD	ADPR	ADPR	ADPR	UBC	UBC/ADPR	EF1	ACT
	0.105	0.078	0.116	0.134	0.146	0.177	0.111	0.218	0.121	0.129	0.197	0.183	0.517
2	ACT	ADPR	UBC	ADPR	ACT	PGD	UBC	CYP	PGD	ACT	CYP	ADPR	ADPR
	0.113	0.079	0.130	0.135	0.150	0.208	0.125	0.229	0.123	0.140	0.206	0.191	0.572
3	ADPR	GAPD	CYP	PGD	EF1	ADPR	ACT	PGD	UBC	ADPR	ACT	PGD	EF1
	0.127	0.090	0.134	0.167	0.177	0.232	0.140	0.271	0.124	0.146	0.227	0.21	0.601
4	PGD	EF1	GAPD	EF1	TUB	CYP	CYP	TBPII	GAPD	GAPD	GAPD	TUB	GAPD
	0.149	0.131	0.339	0.232	0.217	0.262	0.170	0.304	0.190	0.181	0.276	0.339	0.723
5	TBPII	PGD	ADPR	GAPD	GAPD	EF1	EF1	TUB	CYP	CYP	TBPII	ACT	TBPII
	0.168	0.151	0.465	0.394	0.272	0.294	0.217	0.314	0.250	0.220	0.388	0.431	0.949
6	TUB	ACT	ACT	ACT	CYP	ACT	TUB	ACT	EF1	TUB	PGD	CYP	UBC
	0.191	0.175	0.531	0.453	0.430	0.323	0.265	0.369	0.312	0.246	0.481	0.521	1.027
7	GAPD	CYP	TUB	TUB	PGD	UBC	PGD	UBC	ACT	TBPII	EF1	UBC	CYP
	0.212	0.218	0.569	0.511	0.489	0.395	0.384	0.396	0.348	0.352	0.647	0.666	1.106
8	EF1	TUB	TBPII	TBPII	UBC	TBPII	TBPII	GAPD	TUB	PGD	TUB	TBPII	PGD
	0.227	0.267	0.615	0.572	0.531	0.449	0.466	0.495	0.413	0.408	0.772	0.733	1.197
9	CYP	TBPII	EF1	CYP	TBPII	TUB	GAPD	EF1	TBPII	EF1		GAPD	TUB
	0.241	0.314	0.706	0.628	0.571	0.525	0.580	0.579	0.491	0.502		0.806	1.299

**Table 3:** Expression stability values of nine oat candidate reference genes in Mv Pehely calculated by NormFinder. The two types of growing media are indicated with h (hydroponic) and s (soil), while h. is the abbreviation of heavy.

Rank	Osmotic Stress		Salt Stress		H. Metal Stress		Cold Stress			Heat Stress			All Samples
	Leaves (h)	Roots (h)	Leaves (h)	Roots (h)	Leaves (h)	Roots (h)	Leaves (h)	Leaves (s)	Roots (h)	Leaves (h)	Leaves (s)	Roots (h)	
1	PGD	ADPR	GAPD	PGD	CYP	EF1	ADPR	ACT	ADPR	ADPR	ADPR	ADPR	ADPR
	0.096	0.032	0.049	0.090	0.120	0.103	0.121	0.070	0.117	0.091	0.079	0.091	0.178
2	TBPII	GAPD	CYP	ADPR	GAPD	PGD	UBC	CYP	EF1	CYP	UBC	EF1	TBPII
	0.112	0.053	0.196	0.216	0.126	0.108	0.148	0.089	0.142	0.133	0.113	0.169	0.357
3	CYP	PGD	PGD	TBPII	ADPR	CYP	CYP	ADPR	PGD	GAPD	ACT	PGD	UBC
	0.119	0.067	0.218	0.257	0.145	0.191	0.152	0.127	0.146	0.155	0.211	0.215	0.374
4	ADPR	UBC	ADPR	GAPD	ACT	GAPD	ACT	UBC	GAPD	UBC	TBPII	ACT	ACT
	0.124	0.074	0.251	0.268	0.171	0.243	0.216	0.166	0.173	0.173	0.279	0.337	0.400
5	ACT	ACT	ACT	ACT	EF1	ADPR	TUB	TBPII	ACT	ACT	TUB	CYP	EF1
	0.127	0.102	0.276	0.307	0.188	0.247	0.338	0.348	0.178	0.218	0.280	0.406	0.496
6	EF1	TBPII	EF1	UBC	PGD	ACT	GAPD	TUB	UBC	TBPII	GAPD	TBPII	GAPD
	0.137	0.120	0.330	0.308	0.213	0.284	0.357	0.392	0.219	0.383	0.293	0.413	0.588
7	GAPD	CYP	TUB	CYP	TBPII	TBPII	EF1	PGD	TUB	PGD	CYP	TUB	CYP
	0.139	0.138	0.369	0.308	0.270	0.417	0.463	0.408	0.232	0.409	0.332	0.427	0.691
8	TUB	TUB	TBPII	EF1	UBC	UBC	PGD	GAPD	TBPII	EF1	EF1	UBC	TUB
	0.151	0.153	0.382	0.349	0.302	0.444	0.478	0.450	0.241	0.437	0.380	0.694	0.743
9	UBC	EF1	UBC	TUB	TUB	TUB	TBPII	EF1	CYP	TUB	PGD	GAPD	PGD
	0.173	0.157	0.408	0.587	0.354	0.584	0.493	0.492	0.349	0.461	0.507	0.769	0.884

**Table 4:** Expression stability values of nine oat candidate reference genes in Mv Hópehely calculated by NormFinder. The two types of growing media are indicated with h (hydroponic) and s (soil), while h. is the abbreviation of heavy.

Rank	Osmotic Stress		Salt Stress		H. Metal Stress		Cold Stress			Heat Stress			All Samples
	Leaves (h)	Roots (h)	Leaves (h)	Roots (h)	Leaves (h)	Roots (h)	Leaves (h)	Leaves (s)	Roots (h)	Leaves (h)	Leaves (s)	Roots (h)	
1	ACT	GAPD	GAPD	PGD	GAPD	GAPD	ADPR	ADPR	ADPR	ADPR	CYP	ADPR	ADPR
	0.034	0.008	0.137	0.081	0.184	0.055	0.057	0.102	0.090	0.034	0.087	0.041	0.207
2	UBC	UBC	ADPR	EF1	ACT	ADPR	UBC	UBC	PGD	GAPD	ADPR	EF1	ACT
	0.036	0.014	0.243	0.091	0.219	0.110	0.071	0.149	0.125	0.107	0.095	0.052	0.393
3	ADPR	ADPR	ACT	ADPR	ADPR	PGD	ACT	CYP	CYP	UBC	UBC	PGD	TBPII
	0.082	0.021	0.317	0.183	0.253	0.132	0.134	0.163	0.149	0.146	0.137	0.146	0.467
4	PGD	EF1	PGD	UBC	PGD	EF1	CYP	ACT	GAPD	ACT	ACT	TUB	UBC
	0.094	0.025	0.342	0.253	0.278	0.161	0.184	0.174	0.171	0.148	0.208	0.394	0.527
5	GAPD	PGD	CYP	GAPD	CYP	CYP	TUB	PGD	UBC	CYP	GAPD	CYP	EF1
	0.100	0.041	0.344	0.337	0.304	0.223	0.192	0.339	0.188	0.159	0.237	0.401	0.581
6	TUB	ACT	UBC	ACT	UBC	ACT	EF1	TUB	EF1	TUB	TBPII	ACT	GAPD
	0.113	0.072	0.354	0.441	0.336	0.270	0.283	0.370	0.248	0.224	0.512	0.479	0.662
7	TBPII	CYP	TUB	TBPII	EF1	UBC	PGD	TBPII	ACT	PGD	PGD	UBC	CYP
	0.120	0.117	0.385	0.518	0.367	0.349	0.410	0.385	0.282	0.399	0.575	0.583	0.714
8	EF1	TUB/TBPII	TBPII	CYP	TUB	TBPII	TBPII	GAPD	TUB	TBPII	EF1	GAPD	PGD
	0.124	0.175	0.570	0.551	0.431	0.431	0.538	0.395	0.418	0.413	0.609	0.619	0.890
9	CYP		EF1	TUB	TBPII	TUB	GAPD	EF1	TBPII	EF1	TUB	TBPII	TUB
	0.132		0.615	0.638	0.452	0.493	0.617	0.561	0.457	0.528	0.795	0.662	0.945

**Table 5:** Expression stability values of nine oat candidate reference genes in Mv Pehely calculated by Bestkeeper. The two types of growing media are indicated with h (hydroponic) and s (soil), while h. is the abbreviation of heavy.

Rank	Osmotic Stress		Salt Stress		H. Metal Stress		Cold Stress			Heat Stress			All Samples
	Leaves (h)	Roots (h)	Leaves (h)	Roots (h)	Leaves (h)	Roots (h)	Leaves (h)	Leaves (s)	Roots (h)	Leaves (h)	Leaves (s)	Roots (h)	
1	PGD	TBPII	CYP	CYP	UBC	PGD	PGD	TUB	PGD	PGD	TBPII	ADPR	PGD
	0.36 ± 0.08	0.23 ± 0.06	0.58 ± 0.11	0.99 ± 0.19	0.60 ± 0.13	0.66 ± 0.14	0.80 ± 0.17	0.40 ± 0.08	0.67 ± 0.14	0.49 ± 0.11	0.22 ± 0.06	1.16 ± 0.22	1.49 ± 0.32
2	TUB	PGD	TBPII	UBC	TBPII	CYP	TBPII	TBPII	TBPII	TBPII	GAPD	PGD	CYP
	0.52 ± 0.11	0.43 ± 0.09	0.72 ± 0.18	1.00 ± 0.22	0.78 ± 0.19	0.98 ± 0.19	1.06 ± 0.27	0.47 ± 0.12	0.94 ± 0.23	0.75 ± 0.19	0.46 ± 0.09	1.23 ± 0.27	2.44 ± 0.47
3	CYP	UBC	PGD	TBPII	PGD	EF1	UBC	PGD	UBC	UBC	UBC	EF1	TBPII
	0.64 ± 0.13	0.76 ± 0.16	0.79 ± 0.17	1.10 ± 0.27	1.09 ± 0.23	1.15 ± 0.21	1.35 ± 0.30	0.69 ± 0.15	1.14 ± 0.24	1.40 ± 0.31	1.02 ± 0.23	1.49 ± 0.27	2.78 ± 0.69
4	EF1	ADPR	UBC	EF1	CYP	ADPR	ACT	ADPR	EF1	ADPR	PGD	TBPII	UBC
	0.64 ± 0.14	1.03 ± 0.19	1.15 ± 0.26	1.23 ± 0.23	1.67 ± 0.32	1.40 ± 0.26	1.57 ± 0.33	1.25 ± 0.26	1.49 ± 0.28	1.95 ± 0.41	1.13 ± 0.24	1.71 ± 0.41	3.12 ± 0.68
5	TBPII	CYP	GAPD	ADPR	ACT	TBPII	ADPR	ACT	ADPR	GAPD	ADPR	CYP	ADPR
	0.67 ± 0.17	1.32 ± 0.25	1.17 ± 0.24	1.57 ± 0.30	2.33 ± 0.49	1.69 ± 0.40	1.80 ± 0.38	1.66 ± 0.35	1.52 ± 0.29	2.00 ± 0.41	1.40 ± 0.29	2.11 ± 0.40	4.75 ± 0.95
6	GAPD	GAPD	ACT	PGD	EF1	UBC	CYP	CYP	GAPD	CYP	ACT	ACT	ACT
	0.80 ± 0.16	1.35 ± 0.22	2.72 ± 0.57	2.20 ± 0.48	2.40 ± 0.53	2.36 ± 0.50	1.90 ± 0.37	1.97 ± 0.38	1.83 ± 0.30	2.63 ± 0.51	2.93 ± 0.62	2.78 ± 0.50	6.91 ± 1.36
7	ADPR	ACT	ADPR	ACT	ADPR	GAPD	TUB	UBC	CYP	ACT	TUB	TUB	EF1
	1.01 ± 0.22	1.37 ± 0.24	2.77 ± 0.58	4.51 ± 0.83	2.55 ± 0.53	2.43 ± 0.41	3.36 ± 0.71	2.56 ± 0.57	1.85 ± 0.36	2.65 ± 0.56	3.13 ± 0.64	3.11 ± 0.51	7.32 ± 1.48
8	ACT	TUB	EF1	GAPD	GAPD	ACT	GAPD	EF1	ACT	TUB	EF1	UBC	GAPD
	1.05 ± 0.23	1.71 ± 0.28	3.25 ± 0.71	4.68 ± 0.80	2.74 ± 0.56	2.49 ± 0.44	4.31 ± 0.86	4.77 ± 1.04	2.15 ± 0.38	4.33 ± 0.91	4.06 ± 0.90	3.98 ± 0.82	7.88 ± 1.48
9	UBC	EF1	TUB	TUB	TUB	TUB	EF1	GAPD	TUB	EF1	CYP	GAPD	TUB
	1.14 ± 0.26	2.04 ± 0.37	3.57 ± 0.75	7.20 ± 1.24	3.76 ± 0.79	4.46 ± 0.75	4.47 ± 0.96	4.86 ± 0.93	2.37 ± 0.38	4.36 ± 0.94	4.10 ± 0.78	6.04 ± 1.05	9.62 ± 1.84

**Table 6:** Expression stability values of nine oat candidate reference genes in Mv Hópehely calculated by Bestkeeper. The two types of growing media are indicated with h (hydroponic) and s (soil), while h. is the abbreviation of heavy.

Rank	Osmotic Stress		Salt Stress		H. Metal Stress		Cold Stress			Heat Stress			All Samples
	Leaves (h)	Roots (h)	Leaves (h)	Roots (h)	Leaves (h)	Roots (h)	Leaves (h)	Leaves (s)	Roots (h)	Leaves (h)	Leaves (s)	Roots (h)	
1	ADPR	PGD	TBPII	TBPII	TBPII	CYP	PGD	TBPII	PGD	TBPII	TBPII	EF1	PGD
	0.50 ± 0.11	1.21 ± 0.26	0.67 ± 0.17	1.04 ± 0.25	0.62 ± 0.16	0.78 ± 0.15	1.09 ± 0.23	0.30 ± 0.08	0.63 ± 0.13	0.70 ± 0.18	0.45 ± 0.11	0.90 ± 0.16	1.45 ± 0.31
2	TBPII	CYP	PGD	UBC	UBC	PGD	TBPII	PGD	UBC	PGD	PGD	ADPR	CYP
	0.51 ± 0.13	1.45 ± 0.28	0.81 ± 0.17	1.06 ± 0.23	0.99 ± 0.22	1.11 ± 0.24	1.10 ± 0.28	0.35 ± 0.07	1.01 ± 0.22	0.97 ± 0.21	0.64 ± 0.14	0.93 ± 0.17	2.31 ± 0.44
3	PGD	EF1	UBC	PGD	PGD	ADPR	TUB	TUB	ADPR	CYP	GAPD	CYP	TBPII
	0.67 ± 0.14	1.46 ± 0.27	0.83 ± 0.19	1.18 ± 0.25	1.08 ± 0.23	1.36 ± 0.25	1.32 ± 0.28	0.97 ± 0.21	1.07 ± 0.20	1.32 ± 0.26	1.87 ± 0.38	1.11 ± 0.21	2.76 ± 0.69
4	UBC	UBC	CYP	ADPR	CYP	GAPD	UBC	ADPR	CYP	ADPR	UBC	PGD	UBC
	0.72 ± 0.16	1.51 ± 0.33	1.04 ± 0.20	1.28 ± 0.24	1.20 ± 0.23	1.40 ± 0.22	1.99 ± 0.44	1.64 ± 0.35	1.19 ± 0.23	1.61 ± 0.35	2.32 ± 0.51	1.46 ± 0.32	2.78 ± 0.60
5	TUB	GAPDH	GAPD	CYP	GAPD	TBPII	ADPR	CYP	TBPII	GAPD	ADPR	TBPII	ADPR
	0.97 ± 0.21	1.57 ± 0.25	3.01 ± 0.62	1.51 ± 0.28	3.53 ± 0.73	1.82 ± 0.44	2.25 ± 0.48	1.83 ± 0.36	1.40 ± 0.34	1.76 ± 0.37	2.93 ± 0.62	2.58 ± 0.62	5.92 ± 1.19
6	ACT	ADPR	ADPR	EF1	ACT	EF1	ACT	ACT	GAPD	UBC	CYP	UBC	ACT
	0.98 ± 0.22	1.80 ± 0.34	4.00 ± 0.85	2.21 ± 0.41	3.99 ± 0.84	1.89 ± 0.34	2.82 ± 0.60	2.66 ± 0.57	1.73 ± 0.28	1.94 ± 0.43	3.55 ± 0.68	3.21 ± 0.66	7.29 ± 1.44
7	EF1	TBPII	ACT	GAPD	ADPR	UBC	CYP	UBC	EF1	TUB	ACT	TUB	EF1
	1.05 ± 0.25	1.94 ± 0.48	4.69 ± 0.98	4.64 ± 0.78	4.30 ± 0.91	1.91 ± 0.40	2.94 ± 0.57	2.90 ± 0.63	2.17 ± 0.40	2.31 ± 0.49	4.01 ± 0.84	3.32 ± 0.53	8.20 ± 1.69
8	GAPD	ACT	TUB	ACT	EF1	ACT	EF1	GAPD	ACT	ACT	EF1	ACT	GAPD
	1.31 ± 0.28	2.34 ± 0.41	5.07 ± 1.05	4.72 ± 0.84	4.66 ± 1.04	1.97 ± 0.34	3.02 ± 0.69	5.11 ± 1.00	2.58 ± 0.45	2.34 ± 0.50	6.5 ± 1.44	4.16 ± 0.74	8.95 ± 1.69
9	CYP	TUB	EF1	TUB	TUB	TUB	GAPD	EF1	TUB	EF1	TUB	GAPD	TUB
	1.71 ± 0.34	2.85 ± 0.45	6.38 ± 1.41	6.10 ± 1.00	5.24 ± 1.09	3.59 ± 0.57	6.06 ± 1.22	5.14 ± 1.15	3.97 ± 0.64	4.45 ± 1.00	8.19 ± 1.65	5.77 ± 0.98	11.43 ± 2.19

**Table 7:** Comprehensive ranking of nine oat candidate reference genes in Mv Pehely calculated by RefFinder. The two types of growing media are indicated with h (hydroponic) and s (soil), while h. is the abbreviation of heavy.

Rank	Osmotic Stress		Salt Stress		H. Metal Stress			Cold Stress			Heat Stress			All Samples
	Leaves (h)	Roots (h)	Leaves (h)	Roots (h)	Leaves (h)	Roots (h)		Leaves (h)	Leaves (s)	Roots (h)	Leaves (h)	Leaves (s)	Roots (h)	
1	PGD	ADPR	GAPD	ADPR	GAPD		PGD	ADPR	ADPR	ADPR	ADPR	UBC	ADPR	ADPR
2	TBPII	GAPD	CYP	PGD	ADPR		CYP	UBC	ACT	PGD	UBC	TBPII	EF1	TBPII
3	EF1	PGD	PGD	CYP	CYP		EF1	ACT	CYP	GAPD	GAPD	ADPR	PGD	UBC
4	TUB	UBC	ADPR	UBC	ACT		GAPD	CYP	TUB	UBC	CYP	GAPD	ACT	ACT
5	CYP	TBPII	ACT	TBPII	PGD		ACT	PGD	TBPII	EF1	PGD	ACT	CYP	PGD
6	ADPR	ACT	EF1	EF1	UBC		ADPR	TUB	UBC	TBPII	TBPII	TUB	TUB	CYP
7	GAPD	CYP	TBPII	GAPD	TBPII		TBPII	TBPII	PGD	ACT	ACT	CYP	TBPII	EF1
8	ACT	TUB	TUB	ACT	EF1		UBC	GAPD	GAPD	TUB	TUB	PGD	UBC	GAPD
9	UBC	EF1	UBC	TUB	TUB		TUB	EF1	EF1	CYP	EF1	EF1	GAPD	TUB

**Table 8:** Comprehensive ranking of nine oat candidate reference genes in Mv Hópehely calculated by RefFinder. The two types of growing media are indicated with h (hydroponic) and s (soil), while h. is the abbreviation of heavy.

Rank	Osmotic Stress		Salt Stress		H. Metal Stress		Cold Stress			Heat Stress			All Samples
	Leaves (h)	Roots (h)	Leaves (h)	Roots (h)	Leaves (h)	Roots (h)	Leaves (h)	Leaves (s)	Roots (h)	Leaves (h)	Leaves (s)	Roots (h)	
1	ACT	GAPD	GAPD	UBC	ACT	GAPD	ADPR	ADPR	ADPR	ADPR	UBC	ADPR	ADPR
2	UBC	UBC	CYP	PGD	GAPD	PGD	UBC	CYP	PGD	UBC	ADPR	EF1	ACT
3	ADPR	ADPR	PGD	ADPR	ADPR	CYP	ACT	PGD	UBC	GAPD	CYP	PGD	TBPII
4	PGD	EF1	ADPR	EF1	PGD	ADPR	TUB	TBPII	GAPD	ACT	TBPII	CYP	UBC
5	TBPII	PGD	UBC	TBPII	UBC	EF1	PGD	UBC	CYP	CYP	GAPD	TUB	PGD
6	TUB	CYP	ACT	GAPD	CYP	ACT	CYP	TUB	EF1	TBPII	ACT	ACT	CYP
7	GAPD	ACT	TBPII	CYP	TBPII	UBC	EF1	ACT	ACT	PGD	PGD	UBC	EF1
8	EF1	TUB	TUB	ACT	EF1	TBPII	TBPII	GAPD	TBPII	TUB	EF1	TBPII	GAPD
9	CYP	TBPII	EF1	TUB	TUB	TUB	GAPD	EF1	TUB	EF1	TUB	GAPD	TUB

**Table 9:** Primer sequences of nine candidate reference genes used in RT-qPCR analysis.

Gene Abb.	Gene Name	Primer Sequence Forward and Reverse	Amplicon Length (bp)	Efficiency	R^2^	Accession No.
ACT	Actin1	CGAGCGGGAAATTGTAAGGG	191	92%	0.994	MF405765.1
		CGATCATGGATGGCTGGAAG				
TUB	α-Tubulin	AGGTCTTCTCCCGCATCG	90	98%	0.995	U76558.1
		CCTCCTCCATGCCCTCAC				
CYP	Cyclophilin	AGTCCATCTACGGCGAGAAGT	120	93%	0.998	EU035525.1
		GGGACGGTGCAGATGAAGAA				
GAPDH	Glyceraldehyde 3-phosphate dehydrogenase	GTTTGGCATCGTTGAGGGTT	131	93%	0.998	KR029492.1
		TGCTGCTGGGAATGATGTTG				
UBC	Ubiquitin conjugating enzyme	CAAGCTGACCCTGCAATTCA	135	91%	0.992	M62720.1
		GGGCTCCACTGGTTCTGTA				
EF1	Elongation factor 1-α	AAGGAGGCAGCCAACTTCA	122	96%	0.999	M90077.2
		AGCTCAGCAAACTTGACAGC				
TBPII	TATA-binding protein II subunit	GATGAGGCAGCCGAAGATTG	156	91%	0.993	L07604.1
		TCCAAAGTCAACCATCATTGCT				
ADPR	ADP-ribosylation factor	CTCATGGTTGGTCTCGATGC	143	94%	0.998	Ta2291 (Unigene cluster)
		ACATCCCAAACAGTGAAGCT				
PGD	Phosphogluconate dehydrogenase	GCAAAGATGAAACTGGTGGTCA	90	93%	0.995	Ta30797 (Unigene cluster)
		CAACCCACTTTTGTCCGCC				

## Data Availability

The data generated or analysed during this study are included in this published article and its supplementary information files.

## Supplementary Materials

The following are available online at https://www.mdpi.com/article/10.3390/plants10071272/s1, Figures S1–S4: Relative expression of phenylalanine ammonia lyase (PAL) under different abiotic stresses, Figure S5–S14: Sequence aligment and primer location of the investigated genes, Table S1: Sequence homology between wheat (*Triticum aestivum* L.) and the corresponding oat (*Avena sativa* L.) sequences.

## References

[1] Prates L.L., Yu P. (2017). Recent research on inherent molecular structure, physiochemical properties, and bio-functions of food and feed-type Avena sativa oats and processing-induced changes revealed with molecular microspectroscopic techniques. Appl. Spectrosc. Rev..

[2] Butt M.S., Tahir-Nadeem M., Khan M.K.I., Shabir R., Butt M.S. (2008). Oat: Unique among the cereals. Eur. J. Nutr..

[3] Bai J., Yan W., Wang Y., Yin Q., Liu J., Wight C., Ma B. (2018). Screening Oat Genotypes for Tolerance to Salinity and Alkalinity. Front. Plant Sci..

[4] Heuschele D.J., Case A., Smith K.P. (2019). Evaluation of Fast Generation Cycling in Oat (*Avena sativa*). Cereal Res. Commun..

[5] Goff S.A., Ricke D., Lan T.H., Presting G., Wang R., Dunn M., Glazebrook J., Sessions A., Oeller P., Varma H. (2002). A draft sequence of the rice genome (*Oryza sativa* L. ssp. *japonica*). Science.

[6] Yu J., Hu S., Wang J., Wong G.K.S., Li S., Liu B., Deng Y., Dai L., Zhou Y., Zhang X. (2002). A draft sequence of the rice genome (*Oryza sativa* L. ssp. *indica*). Science.

[7] Schnable P.S., Ware D., Fulton R.S., Stein J.C., Wei F., Pasternak S., Liang C., Zhang J., Fulton L., Graves T.A. (2009). The B73 maize genome: Complexity, diversity, and dynamics. Science.

[8] Appels R., Eversole K., Feuillet C., Keller B., Rogers J., Stein N., Pozniak C.J., Choulet F., Distelfeld A., Poland J. (2018). Shifting the limits in wheat research and breeding using a fully annotated reference genome. Science.

[9] Wang Z., Gerstein M., Snyder M. (2009). RNA-Seq: A revolutionary tool for transcriptomics. Nat. Rev. Genet..

[10] Wong M.L., Medrano J.F. (2005). One-Step Versus Two-Step Real- Time PCR. Biotechniques.

[11] Bustin S.A., Benes V., Garson J.A., Hellemans J., Huggett J., Kubista M., Mueller R., Nolan T., Pfaffl M.W., Shipley G.L. (2009). The MIQE Guidelines: Minimum Information for Publication of Quantitative Real-Time PCR Experiments. Clin. Chem..

[12] Pfaffl M.W., Tichopad A., Prgomet C., Neuvians T.P. (2004). Determination of stable housekeeping genes, differentially regulated target genes and sample integrity: BestKeeper—Excel-based tool using pair-wise correlations. Biotechnol. Lett..

[13] Silver N., Best S., Jiang J., Thein S.L. (2006). Selection of housekeeping genes for gene expression studies in human reticulocytes using real-time PCR. BMC Mol. Biol..

[14] Vandesompele J., De Preter K., Pattyn F., Poppe B., Van Roy N., De Paepe A., Speleman F. (2002). Accurate normalization of real-time quantitative RT-PCR data by geometric averaging of multiple internal control genes. Genome Biol..

[15] Andersen C.L., Jensen J.L., Ørntoft T.F. (2004). Normalization of real-time quantitative reverse transcription-PCR data: A model-based variance estimation approach to identify genes suited for normalization, applied to bladder and colon cancer data sets. Cancer Res..

[16] Xie F., Xiao P., Chen D., Xu L., Zhang B. (2012). miRDeepFinder: A miRNA analysis tool for deep sequencing of plant small RNAs. Plant Mol. Biol..

[17] Jarošová J., Kundu J.K. (2010). Validation of reference genes as internal control for studying viral infections in cereals by quantitative real-time RT-PCR. BMC Plant Biol..

[18] Akbarabadi A., Ismaili A., Kahrizi D., Nazarian Firouzabadi F. (2018). Validation of expression stability of reference genes in response to herbicide stress in wild oat (*Avena ludoviciana*). Cell. Mol. Biol..

[19] Liu J., Li P., Lu L., Xie L., Chen X., Zhang B. (2019). Selection and evaluation of potential reference genes for gene expression analysis in avena fatua linn. Plant Prot. Sci..

[20] Yang Z., Wang K., Aziz U., Zhao C., Zhang M. (2020). Evaluation of duplicated reference genes for quantitative real-time PCR analysis in genome unknown hexaploid oat (*Avena sativa* L.). Plant Methods.

[21] Duan Z.L., Han W.H., Yan L., Wu B. (2020). Reference gene selections for real time quantitative PCR analysis of gene expression in different oat tissues and under salt stress. Biol. Plant..

[22] Ding P., Ding Y. (2020). Stories of Salicylic Acid: A Plant Defense Hormone. Trends Plant Sci..

[23] Pawlak-Sprada S., Arasimowicz-Jelonek M., Podgórska M., Deckert J. (2011). Activation of phenylpropanoid pathway in legume plants exposed to heavy metals. Part I. Effects of cadmium and lead on phenylalanine ammonia-lyase gene expression, enzyme activity and lignin content. Acta Biochim. Pol..

[24] Bandurska H., Cieślak M. (2013). The interactive effect of water deficit and UV-B radiation on salicylic acid accumulation in barley roots and leaves. Environ. Exp. Bot..

[25] Li G., Wang H., Cheng X., Su X., Zhao Y., Jiang T., Jin Q., Lin Y., Cai Y. (2019). Comparative genomic analysis of the PAL genes in five *Rosaceae* species and functional identification of Chinese white pear. PeerJ.

[26] Auler P.A., Benitez L.C., do Amaral M.N., Vighi I.L., dos Santos Rodrigues G., da Maia L.C., Braga E.J.B. (2017). Evaluation of stability and validation of reference genes for RT-qPCR expression studies in rice plants under water deficit. J. Appl. Genet..

[27] Paolacci A.R., Tanzarella O.A., Porceddu E., Ciaffi M. (2009). Identification and validation of reference genes for quantitative RT-PCR normalization in wheat. BMC Mol. Biol..

[28] Pu Q., Li Z., Nie G., Zhou J., Liu L., Peng Y. (2020). Selection and Validation of Reference Genes for Quantitative Real-Time PCR in White Clover (*Trifolium repens* L.) Involved in Five Abiotic Stresses. Plants.

[29] Kozera B., Rapacz M. (2013). Reference genes in real-time PCR. J. Appl. Genet..

[30] Joseph J.T., Poolakkalody N.J., Shah J.M. (2019). Screening internal controls for expression analyses involving numerous treatments by combining statistical methods with reference gene selection tools. Physiol. Mol. Biol. Plants.

[31] Zhao J., Zhou M., Meng Y. (2020). Identification and validation of reference genes for rt-qpcr analysis in switchgrass under heavy metal stresses. Genes.

[32] Janská A., Hodek J., Svoboda P., Zámečník J., Prášil I.T., Vlasáková E., Milella L., Ovesná J. (2013). The choice of reference gene set for assessing gene expression in barley (*Hordeum vulgare* L.) under low temperature and drought stress. Mol. Genet. Genom..

[33] Kiarash J.G., Wilde H.D., Amirmahani F., Moemeni M.M., Zaboli M., Nazari M., Moosavi S.S., Jamalvandi M. (2018). Selection and validation of reference genes for normalization of qRT-PCR gene expression in wheat (*Triticum durum* L.) under drought and salt stresses. J. Genet..

[34] Poli M., Salvi S., Li M., Varotto C. (2017). Selection of reference genes suitable for normalization of qPCR data under abiotic stresses in bioenergy crop *Arundo donax* L.. Sci. Rep..

[35] Wang Y., Yu K., Poysa V., Shi C., Zhou Y. (2012). Selection of reference genes for normalization of qRT-PCR analysis of differentially expressed genes in soybean exposed to cadmium. Mol. Biol. Rep..

[36] Zhang L., Zhang Q., Jiang Y., Li Y., Zhang H., Li R. (2018). Reference genes identification for normalization of qPCR under multiple stresses in Hordeum brevisubulatum. Plant Methods.

[37] Pál M., Horváth E., Janda T., Páldi E., Szalai G. (2005). Cadmium stimulates the accumulation of salicylic acid and its putative precursors in maize (*Zea mays*) plants. Physiol. Plant..

[38] Szalai G., Janda K., Darkó É., Janda T., Peeva V., Pál M. (2017). Comparative analysis of polyamine metabolism in wheat and maize plants. Plant Physiol. Biochem..

[39] Türkösi E., Darko E., Rakszegi M., Molnár I., Molnár-Láng M., Cseh A. (2018). Development of a new 7BS.7HL winter wheat-winter barley robertsonian translocation line conferring increased salt tolerance and (1,3;1,4)-β-D-glucan content. PLoS ONE.

[40] Tajti J., Németh E., Glatz G., Janda T., Pál M. (2019). Pattern of changes in salicylic acid-induced protein kinase (SIPK) gene expression and salicylic acid accumulation in wheat under cadmium exposure. Plant Biol..

[41] Hua W., Zhu J., Shang Y., Wang J., Jia Q., Yang J. (2015). Identification of Suitable Reference Genes for Barley Gene Expression Under Abiotic Stresses and Hormonal Treatments. Plant Mol. Biol. Report..

[42] Pál M., Tajti J., Szalai G., Peeva V., Végh B., Janda T. (2018). Interaction of polyamines, abscisic acid and proline under osmotic stress in the leaves of wheat plants. Sci. Rep..

[43] Tajti J., Janda T., Majláth I., Szalai G., Pál M. (2018). Comparative study on the effects of putrescine and spermidine pre-treatment on cadmium stress in wheat. Ecotoxicol. Environ. Saf..

[44] Szalai G., Tajti J., Hamow K.Á., Ildikó D., Khalil R., Vanková R., Dobrev P., Misheva S.P., Janda T., Pál M. (2020). Molecular background of cadmium tolerance in Rht dwarf wheat mutant is related to a metabolic shift from proline and polyamine to phytochelatin synthesis. Environ. Sci. Pollut. Res..

[45] PepsiCo OT3098 Hexaploid Oat Genome Assembly and Annotation Release in Collaboration with GrainGenes. https://web.archive.org/web/20210616152750/https://wheat.pw.usda.gov/GG3/node/922.

[46] Lu S., Wang J., Chitsaz F., Derbyshire M.K., Geer R.C., Gonzales N.R., Gwadz M., Hurwitz D.I., Marchler G.H., Song J.S. (2020). CDD/SPARCLE: The conserved domain database in 2020. Nucleic Acids Res..

[47] Owczarzy R., Tataurov A.V., Wu Y., Manthey J.A., McQuisten K.A., Almabrazi H.G., Pedersen K.F., Lin Y., Garretson J., McEntaggart N.O. (2008). IDT SciTools: A suite for analysis and design of nucleic acid oligomers. Nucleic Acids Res..

[48] Untergasser A., Cutcutache I., Koressaar T., Ye J., Faircloth B.C., Remm M., Rozen S.G. (2012). Primer3-new capabilities and interfaces. Nucleic Acids Res..

[49] Livak K.J., Schmittgen T.D. (2001). Analysis of relative gene expression data using real-time quantitative PCR and the 2-ΔΔCT method. Methods.

