# Supplementary material for: Validation of Reference Genes for Studying Different Abiotic Stresses in Oat (Avena sativa L.) by RT-qPCR
Source: Plants (Basel). 2021 Jun 22;10(7):1272. doi: 10.3390/plants10071272 (PMC8309034; doi:10.3390/plants10071272)
Supplement: Supplementary file 1 [file plants-10-01272-s001.zip › Figure S1-4.pdf]

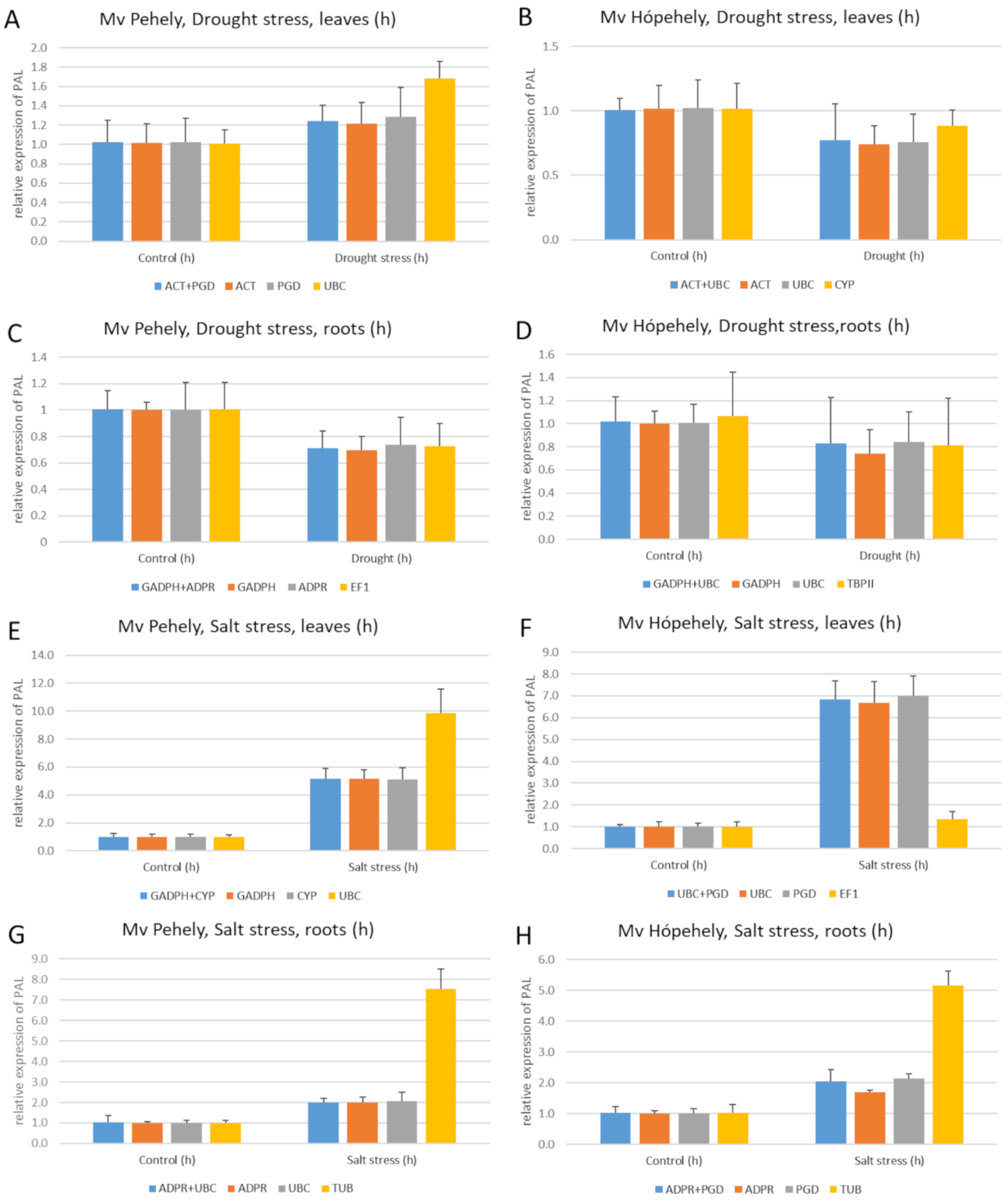

Figure S1. Relative expression of phenylalanine ammonia lyase (PAL) under drought (A-D) and under salt (E-H) stresses. Expression values are normalized by the two most stable and the most unstable reference genes, (h) indicates hydroponic culture.

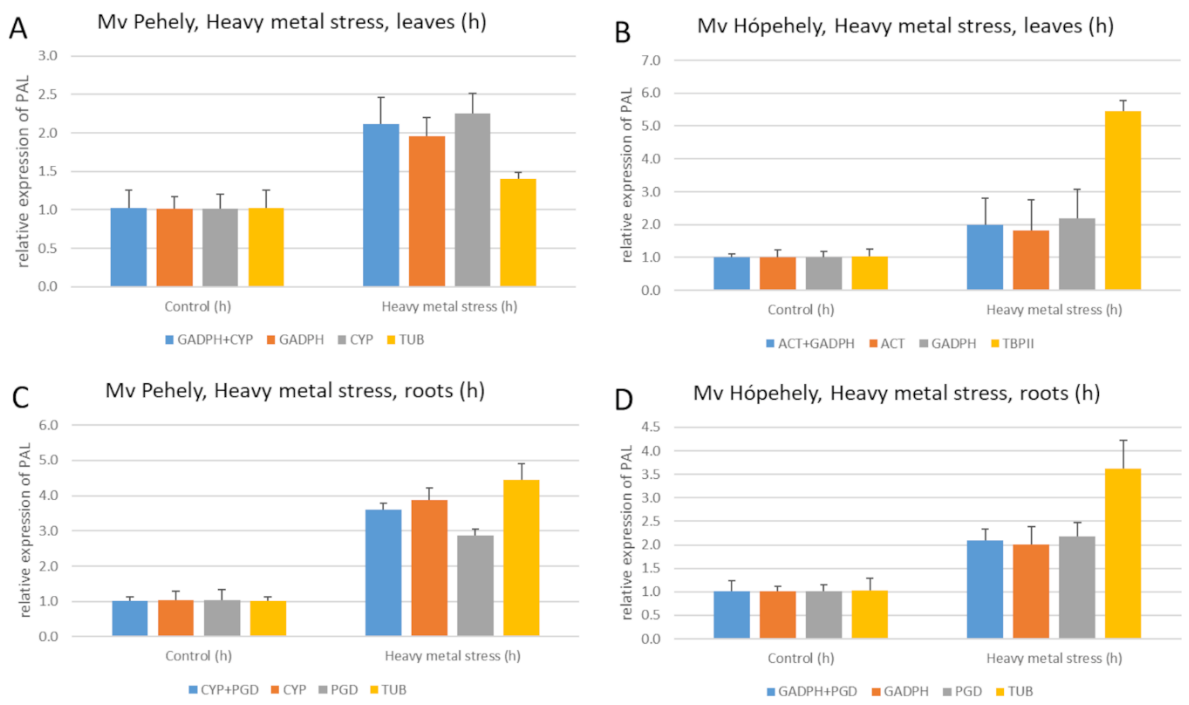

Figure S2. Relative expression of phenylalanine ammonia lyase (PAL) under heavy metal stress (A-D) in the leaves and roots of Mv Pehely and Mv Hópehely. Expression values are normalized by the two most stable and the most unstable reference genes, (h) indicates hydroponic culture.

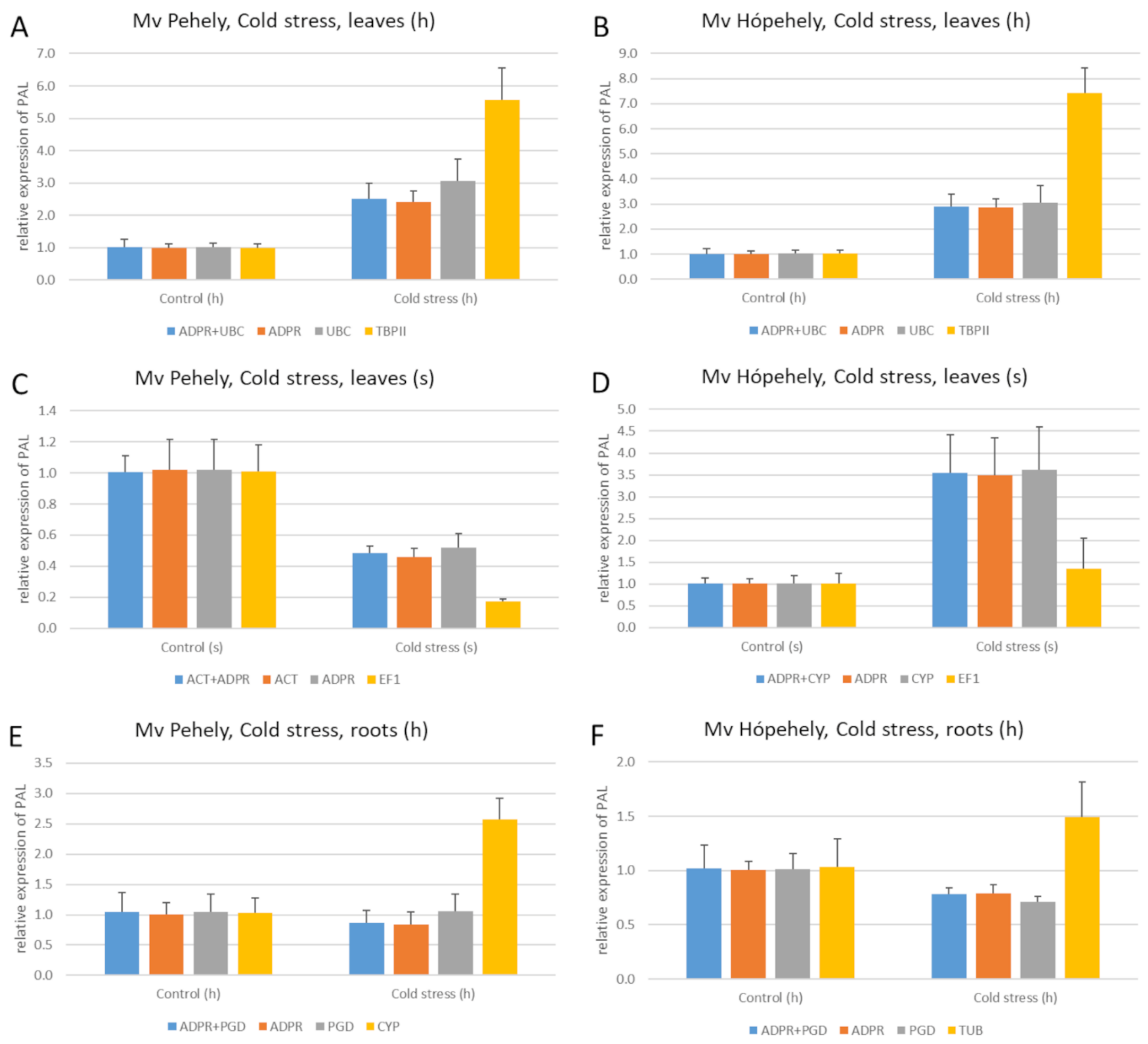

Figure S3. Relative expression of Phenilalanine ammonia lyase (PAL) under cold stress (A-F) in the leaves and roots of Mv Pehely and Mv Hópehely. Expression values are normalized by the two most stable and the most unstable reference genes. The two types of growing media are indicated with h (hydroponic) and s (soil).

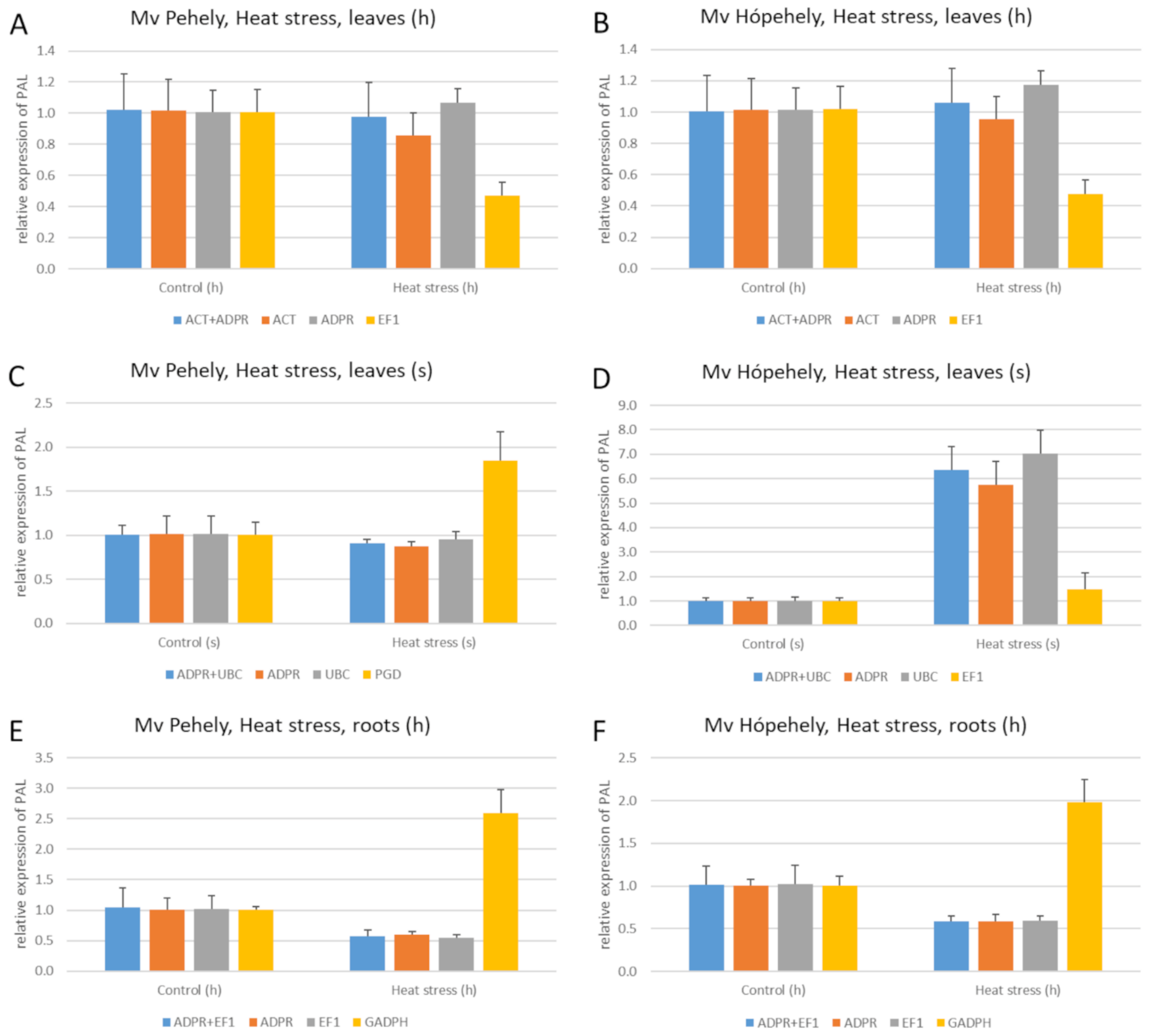

Figure S4. Relative expression of phenylalanine ammonia lyase (PAL) under heat stress (A-F) in the leaves and roots of Mv Pehely and Mv Hópehely. Expression values are normalized by the two most stable and the most unstable reference genes. The two types of growing media are indicated with h (hydroponic) and s (soil).
