# Supplementary material for: Validation of Reference Genes for Studying Different Abiotic Stresses in Oat (Avena sativa L.) by RT-qPCR
Source: Plants (Basel). 2021 Jun 22;10(7):1272. doi: 10.3390/plants10071272 (PMC8309034; doi:10.3390/plants10071272)
Supplement: Supplementary file 1 [file plants-10-01272-s001.zip › Figure S5-14, sequence alignment.pdf]

Actin (ACT)

reference sequence: MF405765.1
forward primer: CGAGCGGGAAATTGTAAGGG
reverse primer: CGATCATGGATGGCTGGAAG

product size: 191 bp
contig number: CTG107765, percentage identity: 95%, E value: 0.00, Gaps (0/615)

```
Query     454  GGTATCGTTCTCGACTCTGGTGATGGTGTCAGCCACACCGTGCCCATTTACGAAGGATAC  513
               ||||| ||||| |||||||||||||||||||||||||| |||||||| || |||||||||
Subject 18164  GGTATTGTTCTTGACTCTGGTGATGGTGTCAGCCACACTGTGCCCATCTATGAAGGATAC  18223

Query     514  GCGCTTCCTCATGCCATTCTTCGTTTGGATCTCGCTGGTCGGGATCTCACGGACTCCCTC  573
               ||||||||||| |||||||||||||||||||| ||||| || |||||||| || || |||
Subject 18224  GCGCTTCCTCACGCCATTCTTCGTTTGGATCTTGCTGGCCGTGATCTCACTGATTCTCTC  18283

Query     574  ATGAAGATCCTCACTGAGAGAGGTTACTCCTTCACAACCTCAGCCGAGCGGGAAATTGTA  633
               |||||||| |||||||||||||||||||||||||||||||||||||||||||||||||||
Subject 18284  ATGAAGATTCTCACTGAGAGAGGTTACTCCTTCACAACCTCAGCCGAGCGGGAAATTGTA  18343

Query     634  AGGGACATCAAGGAGAAGCTTGCGTATGTTGCCCTTGATTATGAACAGGAGCTGGAGACT  693
               |||||||||||||||||||||||||| || ||||||||||||||||| ||||||||||||
Subject 18344  AGGGACATCAAGGAGAAGCTTGCGTACGTAGCCCTTGATTATGAACAAGAGCTGGAGACT  18403

Query     694  GCCAAGAACAGCTCCTCAGTTGAGAAGAGCTACGAGCTTCCTGATGGTCAGGTCATCACG  753
               |||||||||||||||||||||||||||||||||||||| ||||| |||||||| ||||||
Subject 18404  GCCAAGAACAGCTCCTCAGTTGAGAAGAGCTACGAGCTACCTGACGGTCAGGTGATCACG  18463

Query     754  ATTGGCGCAGAGAGGTTCAGGTGCCCTGAGGTCCTCTTCCAGCCATCCATGATCGGCATG  813
               |||||||| |||||||||||||||||||||||||||||||||||||||||||||||||||
Subject 18464  ATTGGCGCTGAGAGGTTCAGGTGCCCTGAGGTCCTCTTCCAGCCATCCATGATCGGCATG  18523

Query     814  GAGTCTTCTGGAATCCATGAGACGACCTACAACTCCATCATGAAGTGTGACGTGGATATC  873
               ||||||||||||||||||||||| ||||||||||||||||||||||||||||||||||||
Subject 18524  GAGTCTTCTGGAATCCATGAGACAACCTACAACTCCATCATGAAGTGTGACGTGGATATC  18583

Query     874  AGGAAGGACCTGTACGGCAACATTGTGCTCAGTGGTGGCACAACTATGTTCCCAGGTATC  933
               |||||||||||||| |||||||||||||||||||| || ||||| |||||||||||||||
Subject 18584  AGGAAGGACCTGTATGGCAACATTGTGCTCAGTGGAGGTACAACCATGTTCCCAGGTATC  18643

Query     934  GCTGACCGTATGAGCAAGGAGATCACCGCGCTTGCTCCGAGCAGCATGAAGATCAAGGTT  993
               ||||||||||||||||||||||||||||||||||||||||||||||||||||||||||||
Subject 18644  GCTGACCGTATGAGCAAGGAGATCACCGCGCTTGCTCCGAGCAGCATGAAGATCAAGGTT  18703

Query     994  GTCGCCCCACCTGAGAGGAAGTACAGTGTCTGGATCGGAGGGTCCATCCTAGCCTCACTC  1053
               || || || |||||||||||||||||||||||||||||||||||||| |||||||| |||
Subject 18704  GTTGCTCCTCCTGAGAGGAAGTACAGTGTCTGGATCGGAGGGTCCATTCTAGCCTCGCTC  18763

Query    1054  AGCACTTTCCAACAG  1068
               |||||||||||||||
Subject 18764  AGCACTTTCCAACAG  18778
```

oat sequence:

```
GGTATTGTTCTTGACTCTGGTGATGGTGTCAGCCACACTGTGCCCATCTATGAAGGATACGCGCTTCCTCACGCCATTCTTCGTTTGGATCTTG
CTGGCCGTGATCTCACTGATTCTCTCATGAAGATTCTCACTGAGAGAGGTTACTCCTTCACAACCTCAGCCGAGCGGGAAATTGTAAGGGACAT
CAAGGAGAAGCTTGCGTACGTAGCCCTTGATTATGAACAAGAGCTGGAGACTSGCCAAGAACAGCTCCTCAGTTGAGAAGAGCTACGAGCTACC
TGACGGTCAGGTGATCACGATTGGCGCTGAGAGGTTCAGGTGCCCTGAGGTCCTCTTCCAGCCATCCATGATCGGCATGGAGTCTTCTGGAATC
CATGAGACAACCTACAACTCCATCATGAAGTGTGACGTGGATATCAGGAAGGACCTGTATGGCAACATTGTGCTCAGTGGAGGTACAACCATGT
TCCCAGGTATCGCTGACCGTATGAGCAAGGAGATCACCGCGCTTGCTCCGAGCAGCATGAAGATCAAGGTTGTTGCTCCTCCTGAGAGGAAGTA
CAGTGTCTGGATCGGAGGGTCCATTCTAGCCTCGCTCAGCACTTTCCAACAG
```

Figure S5. Actin sequence alignment and primer location

α-Tubulin (TUB)

reference sequence: U76558.1

forward primer: AGGTCTTCTCCCGCATCG
reverse primer: CCTCCTCCATGCCCTCAC

product size: 90 bp

contig number: CTG25086, percentage identity: 93%, E value: 0.00, gaps (4/619)

```
Query 10       CAGGTCATTTCATCACTGACAGCTTCCCTGAGGTTTGATGGTGCTCTGAATGTTGATGTC 69
               ||||||||||||||  |||| |||||||||||||||||||||||||| || |||||||||
Subject 140098 CAGGTCATTTCATCGTTGACTGCTTCCCTGAGGTTTGATGGTGCTCTTAACGTTGATGTC 140157

Query 70       AATGAATTCCAGACCAACTTGGTGCCCTACCCGAGGATCCACTTCATGCTTTCCTCCTAT 129
               ||||| |||||||||||  ||||||||||||| |||||||||||||||||| ||||||| 
Subject 140158 AATGAGTTCCAGACCAATCTGGTGCCCTACCCAAGGATCCACTTCATGCTCTCCTCCTAC 140217

Query 130      GCCCCAGTGATCTCAGCTGAGAAGGCTTACCATGAGCAGCTGTCCGTTGCTGAGATCACC 189
               |||||||||||||| ||||||||||||||||| |||||||| || |||||||||||||||
Subject 140218 GCCCCAGTGATCTCTGCTGAGAAGGCTTACCACGAGCAGCTCTCTGTTGCTGAGATCACC 140277

Query 190      AACAGCGCCTTTGAGCCTTCGTCCATGATGGCCAAGTGCGACCCCCGCCACGGCAAGTAC 249
               ||||| |||||||||||||| ||||||||||||||||||||||||||||| |||||||||
Subject 140278 AACAGTGCCTTTGAGCCTTCCTCCATGATGGCCAAGTGCGACCCCCGCCATGGCAAGTAC 140337

Query 250      ATGGCCTGCTGTCTCATGTACCGTGGTGATGTTGTGCCAAAGGACGTCAACGCTGCTGTG 309
               |||||||||||||| ||||||||||||||||| ||||| ||||| || || ||  |||||
Subject 140338 ATGGCCTGCTGTCTGATGTACCGTGGTGATGTGGTGCCCAAGGATGTGAATGC--CTGTG 140395

Query 310      GCCACCATCAAGACCAAGCGCACTATTCAGTTTGTTGACTGGTGCCCCACTGGCTTCAAG 369
               |||||||||||||||||||||||||| |||||||| ||||||||||||||||||||||||
Subject 140396 GCCACCATCAAGACCAAGCGCACTATCCAGTTTGTGGACTGGTGCCCCACTGGCTTCAAG 140455

Query 370      TGTGGTA-TCAACTACCAGCCACCAGGTGTCGTCCCAGGCGGTGACCTTGCCAAGGTCCA 428
               || |||| ||||||||||||||||  | || |||||||| ||||||||||||||||||||
Subject 140456 TGCGGTATTCAACTACCAGCCACCCAGCGTTGTCCCAGGTGGTGACCTTGCCAAGGTCCA 140515

Query 429      GAGGGCTGTGTGCATGATCTCCAACTCCACCAGTGTCGTCGAGGTCTTCTCCCGCATCGA 488
               |||||||| ||||||||||||||||||||||||||| || ||||||||||||||||||||
Subject 140516 GAGGGCTG-GTGCATGATCTCCAACTCCACCAGTGTTGTTGAGGTCTTCTCCCGCATCGA 140574

Query 489      CCACAAGTTTGACCTGATGTACGCCAAGCGTGCCTTCGTCCACTGGTACGTCGGTGAGGG 548
               ||||||||| ||||||||||| |||||||||||||| |||||||||||||| ||||||||
Subject 140575 CCACAAGTTCGACCTGATGTATGCCAAGCGTGCCTTTGTCCACTGGTACGTGGGTGAGGG 140634

Query 549      CATGGAGGAGGGAGAGTTCTCTGAGGCCCGTGAGGATCTCGCTGCCCTGGAGAAGGACTA 608
                |||||||||||||||||||||||||||||||||||||| ||||||||||||||||||||
Subject 140635 TATGGAGGAGGGAGAGTTCTCTGAGGCCCGTGAGGATCTTGCTGCCCTGGAGAAGGACTA 140694

Query 609      TGAAGAAGTTGGTGCTGAG 627
               |||||||||||||||||||
Subject 140695 TGAAGAAGTTGGTGCTGAG 140713
```

oat sequence:

CAGGTCATTTCATCGTTGACTGCTTCCCTGAGGTTTGATGGTGCTCTTAACGTTGATGTCAATGAGTTCCAGACCAATCTGGTGCCCTACCCAA
GGATCCACTTCATGCTCTCCTCCTACGCCCCAGTGATCTCTGCTGAGAAGGCTTACCACGAGCAGCTCTCTGTTGCTGAGATCACCAACAGTGC
CTTTGAGCCTTCCTCCATGATGGCCAAGTGCGACCCCCGCCATGGCAAGTACATGGCCTGCTGTCTGATGTACCGTGGTGATGTGGTGCCCAAG
GATGTGAATGCCTGTGGCCACCATCAAGACCAAGCGCACTATCCAGTTTGTGGACTGGTGCCCCACTGGCTTCAAGTGCGGTATTCAACTACCA
GCCACCCAGCGTTGTCCCAGGTGGTGACCTTGCCAAGGTCCAGAGGGCTGGTGCATGATCTCCAACTCCACCAGTGTTGTTGAGGTCTTCTCCC
GCATCGACCACAAGTTCGACCTGATGTATGCCAAGCGTGCCTTTGTCCACTGGTACGTGGGTGAGGGTATGGAGGAGGGAGAGTTCTCTGAGGC
CCGTGAGGATCTTGCTGCCCTGGAGAAGGACTATGAAGAAGTTGGTGCTGAG

Figure S6. α-Tubulin sequence alignment and primer location

Cyclophilin (CYP)

reference sequence: EU035525.1

forward primer: AGTCCATCTACGGCGAGAAGT

reverse primer: AGATGAAGAACTGGGAGCCG

117 bp

sequence identity 92%, E-value: 0.00, gaps: 0/498, contig number: CTG81232

sequence alignment (*T.aestivum=query* and *A. sativa=subject)*

```
Query        1  CCGAAGGTGTTCTTCGACATGACGGTCGGCGGCGCGCCGGCGGGGCGCATCGTGATGGAG  60
                || ||||| |||||||||||||| ||||||||||  ||||| || ||||||| |||||||
Subject  13074  CCCAAGGTCTTCTTCGACATGACCGTCGGCGGCGTCCCGGCAGGCCGCATCGAGATGGAG  13015

Query       61  CTGTACAAGGACGCGGTGCCGAGGACGGTGGAGAACTTCCGCGCGCTGTGCACGGGCGAG  120
                |||| |  | ||| ||| ||  | |||| |||||||||||||||||||||||| ||||||
Subject  13014  CTGTTCGCGAACGAGGTCCCCCGCACGGCGGAGAACTTCCGCGCGCTGTGCACCGGCGAG  12955

Query      121  AAGGGCGTGGGCAAGAGCGGCAAGCCGCTGCACTACAAGGGGAGCGCGTTCCACCGCGTG  180
                ||||||||||||||||||||||||||||||||||||||||| ||| ||||||||||||||
Subject  12954  AAGGGCGTGGGCAAGAGCGGCAAGCCGCTGCACTACAAGGGCAGCTCGTTCCACCGCGTG  12895

Query      181  ATCCCCGACTTCATGTGCCAGGGCGGCGACTTCACCAGGGGCAACGGGACGGGCGGCGAG  240
                ||||||||||||||||||||||||||||||||||||| ||||||||| ||||||||||||
Subject  12894  ATCCCCGACTTCATGTGCCAGGGCGGCGACTTCACCAAGGGCAACGGCACGGGCGGCGAG  12835

Query      241  TCCATCTACGGCGAGAAGTTCGCCGACGAGAAGTTCGTCCACAAGCACACCAAGCCCGGG  300
                ||||||||||||||||||||||||||||||  ||||||| | ||||||||||||||||||
Subject  12834  TCCATCTACGGCGAGAAGTTCGCCGACGAGCGGTTCGTCAAGAAGCACACCAAGCCCGGG  12775

Query      301  ATCCTCTCCATGGCCAACGCCGGGCCCAACACCAACGGCTCCCAGTTCTTCATCTGCACC  360
                ||||||||||||||||||||||||||||||||||||||||||||||||||||||||||||
Subject  12774  ATCCTCTCCATGGCCAACGCCGGGCCCAACACCAACGGCTCCCAGTTCTTCATCTGCACC  12715

Query      361  GTCCCCTGCAATTGGCTCGACGGCAAGCACGTCGTCTTCGGCGAGGTCGTCGAGGGCATG  420
                |||||||||   ||||||||||||||||||||||||||||||||||||  | ||||||||
Subject  12714  GTCCCCTGCGCCTGGCTCGACGGCAAGCACGTCGTCTTCGGCGAGGTCACCAAGGGCATG  12655

Query      421  GACGTCGTCAAGAACATCGAGAAGGTGGGCTCCCGCAGCGGCACCACCGCCAAGCAGGTC  480
                ||||||||||||  ||||||||||||||| |||||||||||| |||||||||||   |||
Subject  12654  GACGTCGTCAAGGCCATCGAGAAGGTGGGATCCCGCAGCGGCTCCACCGCCAAGGCCGTC  12595

Query      481  GTCATCGCCGACTGCGGC  498
                ||||||||||||||||||
Subject  12594  GTCATCGCCGACTGCGGC  12577
```

oat sequence:

CCCAAGGTCTTCTTCGACATGACCGTCGGCGGCGTCCCGGCAGGCCGCATCGAGATGGAGCTGTTCGCGAACGAGGTCCCCCGCACGGCGGAGAACTTCCGCGCGCT
GTGCACCGGCGAGAAGGGCGTGGGCAAGAGCGGCAAGCCGCTGCACTACAAGGGCAGCTCGTTCCACCGCGTGATCCCCGACTTCATGTGCCAGGGCGGCGACTTCA
CCAAGGGCAACGGCACGGGCGGCGAGTCCATCTACGGCGAGAAGTTCGCCGACGAGCGGTTCGTCAAGAAGCACACCAAGCCCGGGATCCTCTCCATGGCCAACGCC
GGGCCCAACACCAACGGCTCCCAGTTCTTCATCTGCACCGTCCCCTGCGCCTGGCTCGACGGCAAGCACGTCGTCTTCGGCGAGGTCACCAAGGGCATGGACGTCGT
CAAGGCCATCGAGAAGGTGGGATCCCGCAGCGGCTCCACCGCCAAGGCCGTCGTCATCGCCGACTGCGGC

Figure S7. α-Tubulin sequence alignment and primer location

Glyceraldehyde 3-phosphate dehydrogenase (GAPDH)

reference sequence: KR029492.1

forward primer: GTTTGGCATCGTTGAGGGTT
reverse primer: TGCTGCTGGGAATGATGTTG

product size: 131 bp

contig number: ctg108160, percentage identity: ≥87%, E value: ≤$6.38*10^{-18}$

E value: $6.38*10^{-18}$, percentage identity: 95%, gaps 0/61

```
Query 491     AGGTTATCAATGACAGGTTTGGCATTGTTGAGGGTTTGATGACCACAGTTCATGCCATGAC 551
              ||||||||||||||| ||||||||| |||||||||||||||||||| ||||||||||||||
Subject 18579 AGGTTATCAATGACAAGTTTGGCATCGTTGAGGGTTTGATGACCACTGTTCATGCCATGAC 18639
```

E value: $2.05*10^{-27}$, Identity: 89%, gaps 3/104

```
Query 550     ACTGCAACCCAGAAGACTGTTGATGGTCCTTCCAGCAAGGACTGGAGAGGTGGAAGGGCT 609
              || |||||||||||||||||||||| ||| || |||||| ||||||||||||||||||||
Subject 13442 ACAGCAACCCAGAAGACTGTTGATG-TCCCTCGAGCAAG-ACTGGAGAGGTGGAAGGGCT 13499

Query 610     GCCAGCTT-CAACATCATTCCAAGCAGCACTGGTGCCGCAAAGG 652
              || ||||| |||||||||||| ||||||||||| || || ||||
Subject 13500 GCTAGCTTTCAACATCATTCCCAGCAGCACTGGAGCTGCTAAGG 13543
```

E value: $3.67*10^{-39}$, Identity: 87%, gaps 4/145

```
Query 652     GCTGTTGGCAAGGTGCTCCCAGAGCTTAA-CGGAAAGTTGACTGGAATGGCCTTCCGTGT 710
              ||||||||||||||||| ||||  ||||| |||||||||||| |||||||||||||||||
Subject 13670 GCTGTTGGCAAGGTGCTTCCAGTCCTTAAACGGAAAGTTGACCGGAATGGCCTTCCGTGT 13729

Query 711     TCCCACTGTTGATGTTTCTGTTGTTGATCTGACTGTTAGACTTGCGAAGCCAGCAACCTA 770
               || ||||| |||||||||||| |||||||||| |||||||||| | || |||| |||||
Subject 13730 CCCAACTGTCGATGTTTCTGTT-TTGATCTGACCGTTAGACTTGAG-AGGCAGCCACCTA 13787

Query 771     TGATCAGATTAAGGCTGCTATCAAG 795
              ||| ||||| |||||| | ||||||
Subject 13788 TGAGCAGATCAAGGCT-CGATCAAG 13811
```

oat sequence:

```
AGGTTATCAATGACAAGTTTGGCATCGTTGAGGGTTTGATGACCACTGTTCATGCCATGACACAGCAACCCAGAAGACTGTTGATGTCCCTCGA
GCAAGACTGGAGAGGTGGAAGGGCTGCTAGCTTTCAACATCATTCCCAGCAGCACTGGAGCTGCTAAGGCTGTTGGCAAGGTGCTTCCAGTCCTTAAA
CGGAAAGTTGACCGGAATGGCCTTCCGTGTCCCAACTGTCGATGTTTCTGTTTTGATCTGACCGTTAGACTTGAGAGGCAGCCACCTATGAGCAGATCAAGGCTCGA
TCAAG
```

Figure S8. GAPDH sequence alignment and primer location

Ubiquitin conjugating enzyme (UBC)

reference sequence: M62720.1

forward primer: CAAGCTGACCCTGCAATTCA

reverse primer: GGGCTCCACTGGTTCTGTA

product size: 135 bp

contig number: CTG29477, percentage identity: ≥93%, E value: ≤1.74 x 10^{-30}

```
E value 2.75 × 10-34      Identities: 96% Gaps: 0/92

Query     132  AGGTACGTTCAAGCTGACCCTGCAATTCACAGAAGATTACCCCAACAAGCCACCAACTGT  191
               ||||||||||||||||||||||||||||||||||||||| |||||||| |||||||||||
Subject 65992  AGGTACGTTCAAGCTGACCCTGCAATTCACAGAAGATTATCCCAACAAACCACCAACTGT  65933

Query     192  TCGGTTTGTCTCAAGGATGTTTCACCCAAACANN  223
               ||||||||||||||||||||||||||| ||||
Subject 65932  TCGGTTTGTCTCAAGGATGTTTCACCCGAACA  65901


E value 1.74 × 10-30      Identities: 95% Gaps: 0/87

Query     226  TATGCAGATGGAAGCATCTGCTTGGACATCCTACAGAACCAGTGGAGCCCTATATATGAT  285
               |||||||||||||||||||||||||| ||| ||||||||||||||||||| |||||||||
Subject 65783  TATGCAGATGGAAGCATCTGCTTGGATATCTTACAGAACCAGTGGAGCCCCATATATGAT  65724

Query     286  GTTGCAGCAATATTGACCTCTATCCAG  312
               ||||||||||||||||||||| |||||
Subject 65723  GTTGCAGCAATATTGACCTCTGTCCAG  65697


E value 1.52 × 10-37      Identities: 93% Gaps: 0/108

Query 310     CAGTCCCTGCTGTGCGACCCGAACCCGAATTCTCCTGCGAACTCTGAAGCAGCCAGAATG 369
              ||||||||||||||||||||||| || |||||||| |||||||||||||||||||| |||
Subject 65027 CAGTCCCTGCTGTGCGACCCGAATCCAAATTCTCCGGCGAACTCTGAAGCAGCCAGGATG 64968

Query     370 TACAGCGAGAACAAGCGCGAGTACAACCGCAAGGTTCGTGAGGTCGTG 417
              || ||||||||||||||||||||||||||||| ||||||||| |||||
Subject 64967 TATAGCGAGAACAAGCGCGAGTACAACCGCAAAGTTCGTGAGATCGTG 64920


oat sequence:
AGGTACGTTCAAGCTGACCCTGCAATTCACAGAAGATTATCCCAACAAACCACCAACTGTTCGGTTTGTCTCAAGGATGTTTCACCCGAACANN
TATGCAGATGGAAGCATCTGCTTGGATATCTTACAGAACCAGTGGAGCCCCATATATGATGTTGCAGCAATATTGACCTCTGTCCAGTCCCTGC
TGTGCGACCCGAATCCAAATTCTCCGGCGAACTCTGAAGCAGCCAGGATGTATAGCGAGAACAAGCGCGAGTACAACCGCAAAGTTCGTGAGAT
CGTG
```

Figure S9. UBC sequence alignment and primer location

Translation Elongation Factor 1 alpha-subunit

reference sequence: M90077.2

forward primer AAGGAGGCAGCCAACTTCA

reverse primer AGCTCAGCAAACTTGACAGC

122 bp

contig number: CTG31755

E value 0.00 Identities:: 92% Gaps: 2/938

```
Query        1  AGATGGACGCCACCACTCCCAAGTACTCAAAGGCGCGTTATGAAGAAATTGTCAAGGAGG  60
                ||||||| |||||||||||||||||||| ||||| ||||||||||||||||| ||||| |
Subject 21225  AGATGGATGCCACCACTCCCAAGTACTCGAAGGCACGTTATGAAGAAATTGTGAAGGAAG  21284

Query       61  TCTCTTCCTACCTGAAGAAGGTCGGCTACAACCCTGACAAGGTTCCCTTCGTCCCCATCT  120
                ||||||| |||||||||||||| |||||||||||||||||||| || || ||||||||||
Subject 21285  TCTCTTCTTACCTGAAGAAGGTTGGCTACAACCCTGACAAGGTGCCATTTGTCCCCATCT  21344

Query      121  CTGGGTTTGAGGGTGACAACATGATTGAGAGGTCCACCAACCTTGACTGGTACAAGGGCC  180
                |||||||||||||||||||||||||||||||||| |||||||||||||||||||||||||
Subject 21345  CTGGGTTTGAGGGTGACAACATGATTGAGAGGTCAACCAACCTTGACTGGTACAAGGGCC  21404

Query      181  CAACCCTTCTTGAGGCGCTTGACCAGATCAACGAGCCCAAGAGGCCCTCAGACAAGCCCC  240
                | ||| | ||||||||||||||||||||||||||||||||||||||||||||||||||||
Subject 21405  CCACCTTGCTTGAGGCGCTTGACCAGATCAACGAGCCCAAGAGGCCCTCAGACAAGCCCC  21464

Query      241  TCCGTCTTCCCCTCCAGGACGTTTACAAGATTGGTGGCATTGGAACTGTGCCTGTTGGCC  300
                ||||||||||||||||||| ||||||||||||||||||||||||||||||||||||||||
Subject 21465  TCCGTCTTCCCCTCCAGGATGTTTACAAGATTGGTGGCATTGGAACTGTGCCTGTTGGCC  21524

Query      301  GTGTTGAGACTGGTGTCATCAAGCCTGGTATGGTTGTCACCTTTGGTCCCACTGGTCTGA  360
                |||||||||| ||||||||||||||||| |||||||||||||||||||| ||||||||||
Subject 21525  GTGTTGAGACCGGTGTCATCAAGCCTGGCATGGTTGTCACCTTTGGTCCAACTGGTCTGA  21584

Query      361  CAACTGAGGTCAAGTCCGTTGAGATGCACCATGAGTCTCTCCTGGAGGCGCTTCCTGGTG  420
                | |||||||| |||||||||||||||||||| |||||||||||||||||||| |||||||
Subject 21585  CCACTGAGGTTAAGTCCGTTGAGATGCACCACGAGTCTCTCCTGGAGGCGCTCCCTGGTG  21644

Query      421  ACAACGTTGGCTTCAATGTCAAGAATGTTGCCGTGAAGGATCTGAAGCGTGGTTTTGTTG  480
                ||||||| || |||||||||||||||||||| ||||||||||| |||||||| ||||| |
Subject 21645  ACAACGTCGGGTTCAATGTCAAGAATGTTGCTGTGAAGGATCTCAAGCGTGGGTTTGTGG  21704

Query      481  CATCCAACTCCAAGGATGACCCTGCCAAGGAGGCAGCCAACTTCACCTCCCAGGTCATCA  540
                | ||||||||||||||||||||||| ||||||||||||||||||||||||||||||||||
Subject 21705  CGTCCAACTCCAAGGATGACCCTGCAAAGGAGGCAGCCAACTTCACCTCCCAGGTCATCA  21764

Query      541  TCATGAACCACCCTGGTCAGATTGGCAACGGCTACGCCCCAGTGCTGGACTGCCACACCT  600
                |||||||||||||||||||||| ||||||||||| ||||||||||||||||||||||| |
Subject 21765  TCATGAACCACCCTGGTCAGATAGGCAACGGCTATGCCCCAGTGCTGGACTGCCACACTT  21824

Query      601  CGCACATTGCTGTCAAGTTTGCTGAGCTGGTGACCAAGATCGACAGGCGATCTGGTAAGG  660
                |||||||||||||||||||||||||||||||||||||||| ||||| |||||||| ||||
Subject 21825  CGCACATTGCTGTCAAGTTTGCTGAGCTGGTGACCAAGATTGACAGACGATCTGGCAAGG  21884

Query      661  AGCTGGAGGCCCTGCCCAAGTTCCTCAAGAATGGTGATGCTGGCATAGTGAAGATGATTC  720
                || | |||     ||||||||| || ||||||||||||||||| ||||||||||||||||
Subject 21885  AGATTGAGAAGGAGCCCAAGTTTCTGAAGAATGGTGATGCTGGGATAGTGAAGATGATTC  21944

Query      721  CCACCAAGCCCATGGTTGTGGAGACCTTTGCTACTTACCCACCTCTTGGTCGTTTTGCTG  780
                ||||||||||||||||||||||||||||| |||||||||| ||||||||||| |||||||
Subject 21945  CCACCAAGCCCATGGTTGTGGAGACCTTTTCTACTTACCCTCCTCTTGGTCGCTTTGCTG  22004

Query      781  TCCGTGACATGAGGCAAACTGTGGCTGTTGGTGTCATCAAGGGTGTGGAGAAGAAGGACC  840
                | ||||||||||| ||||| || |||||||||||||||||||  ||||||||||||||||
Subject 22005  TGCGTGACATGAGACAAACGGTTGCTGTTGGTGTCATCAAGGCCGTGGAGAAGAAGGACC  22064

Query      841  CAACCGGCGCCAAGGTGACCAAGGCTGCCATCAAGAAGAAATGAGGGGCTTACCTGAATC  900
                | ||||| ||||||||||||||||||||   ||||||||||||||| | ||||||  |||
Subject 22065  CTACCGGTGCCAAGGTGACCAAGGCTGCTGCCAAGAAGAAATGAGGTGTTTACCT-TATC  22123

Query      901  CATCTATGTCTGCTTTTGAGAT-CTCTAGTAGTTTAGT  937
                  || ||||||| ||||||||| | |||||||||| ||
Subject 22124  GTTCCATGTCTGGTTTTGAGATACCCTAGTAGTTTTGT  22161
```

oat sequence:

AGATGGATGCCACCACTCCCAAGTACTCGAAGGCACGTTATGAAGAAATTGTGAAGGAAGTCTCTTCTTACCTGAAGAAGGTTGGCTACAACCCTGACAAGGTGCCATTTGTCCCCATCTCTGGG
TTTGAGGGTGACAACATGATTGAGAGGTCAACCAACCTTGACTGGTACAAGGGCCCCACCTTGCTTGAGGCGCTTGACCAGATCAACGAGCCCAAGAGGCCCTCAGACAAGCCCCTCCGTCTTCC
CCTCCAGGATGTTTACAAGATTGGTGGCATTGGAACTGTGCCTGTTGGCCGTGTTGAGACCGGTGTCATCAAGCCTGGCATGGTTGTCACCTTTGGTCCAACTGGTCTGACCACTGAGGTTAAGT
CCGTTGAGATGCACCACGAGTCTCTCCTGGAGGCGCTCCCTGGTGACAACGTCGGGTTCAATGTCAAGAATGTTGCTGTGAAGGATCTCAAGCGTGGGTTTGTGGCGTCCAACTCCAAGGATGAC
CCTGCAAAGGAGGCAGCCAACTTCACCTCCCAGGTCATCATCATGAACCACCCTGGTCAGATAGGCAACGGCTATGCCCCAGTGCTGGACTGCCACACTTCGCACATTGCTGTCAAGTTTGCTGA
GCTGGTGACCAAGATTGACAGACGATCTGGCAAGGAGATTGAGAAGGAGCCCAAGTTTCTGAAGAATGGTGATGCTGGGATAGTGAAGATGATTCCCACCAAGCCCATGGTTGTGGAGACCTTTT
CTACTTACCCTCCTCTTGGTCGCTTTGCTGTGCGTGACATGAGACAAACGGTTGCTGTTGGTGTCATCAAGGCCGTGGAGAAGAAGGACCCTACCGGTGCCAAGGTGACCAAGGCTGCTGCCAAG
AAGAAATGAGGTGTTTACCT-TATCGTTCCATGTCTGGTTTTGAGATACCCTAGTAGTTTTGT

Figure S10. EF1 sequence alignment and primer location

TATA binding protein II subunit (TBPII)

reference sequence: L07604.1

forward primer: GATGAGGCAGCCGAAGATTG
reverse primer: TCCAAAGTCAACCATCATTGCT

product size: 156 bp

contig number: CTG9379, percentage identity: ≥90%, E value: ≤$5.56*10^{-07}$

```
E value: 3.75*10-09, Identity: 92%, gaps 0/48

Query      224  AGTATGCTCGTATAATTCAGAAGCTTGGATTTCCTGCAAAATTCAAGG  271
                |||||||||||||||| |||||||||   |||||||||||||||||||
Subject 243738  AGTATGCTCGTATAATCCAGAAGCTTAACTTTCCTGCAAAATTCAAGG  243691

E value: 31.39*10-33, Identity: 93%, gaps 0/101

Query      263 AATTCAAGGACTTCAAGATCCAGAATATTGTTGCCTCTTGTGATGTCAAATTCCCTATCA 322
               ||| |||||||||||||||||||||||||||||| ||||||||||||||||||||||| |
Subject 243608 AATACAAGGACTTCAAGATCCAGAATATTGTTGCATCTTGTGATGTCAAATTCCCTATAA 243549

Query      323 GGCTTGAGGGCCTCGCATATTCCCATGGCGCTTTCTCAAGT 363
               ||||||||||||| || ||||| |||||||||||||| |||
Subject 243548 GGCTTGAGGGCCTGGCTTATTCTCATGGCGCTTTCTCCAGT 243508

E value: 3.28*10-35, Identity: 93%, gaps 0/104

Query 364      TATGAGCCAGAGCTATTTCCCGGTCTGATTTACCGGATGAGGCAGCCGAAGATTGTCCTC 423
               |||||||||||||||||||| |||||||| || ||||||||||||||||||||||||||
Subject 243380 TATGAGCCAGAGCTATTTCCTGGTCTGATCTATCGGATGAGGCAGCCGAAGATTGTCCTG 243321

Query 424      CTAATTTTTGTTTCTGGCAAGATTGTTCTGACTGGAGCAAAGGT 467
               ||||||||||| || ||||||||||||||||||||||| |||||
Subject 243320 CTAATTTTTGTCTCGGGCAAGATTGTTCTGACTGGAGCGAAGGT 243277

E value: 1.22*10-21, Identity: 90%, gaps 0/81

Query      464 AGGTGAGAGAAGAGACATACTCCGCCTTCGAGAACATATATCCTGTCCTGACAGAGTTCA 523
               ||||||||||| |||| || | |||||||||||||||||||| ||||| ||||||||||
Subject 243205 AGGTGAGAGAACAGACGTATACTGCCTTCGAGAACATATATCCCGTCCTCACAGAGTTCA 243146

Query 524      GAAAAGTCCAGCAATGATGGT 544
               ||||||| |||||||||||||
Subject 243145 GAAAAGTTCAGCAATGATGGT 243125

E value: 5.56*10-07, Identity: 91%, gaps 1/46

Query 544      TGACTTTGGAAGTAAAGCAGAATTGGAGACATTGTTGCTTGCTGCC 589
               |||||||||||||||| |||||||||||||| ||||||||||| |||
Subject 242927 TGACTTTGGAAGTAA-GCAGAATTGGAGATGTTGTTGCTTGCGGCC 242883
```

oat sequence:

AGTATGCTCGTATAATCCAGAAGCTTAACTTTCCTGCAAAATTCAAGGACTTCAAGATCCAGAATATTGTTGCATCTTGTGATGTCAAATTCCC
TATAAGGCTTGAGGGCCTGGCTTATTCTCATGGCGCTTTCTCCAGTATGAGCCAGAGCTATTTCCTGGTCTGATCTATCGGATGAGGCAGCCGA
AGATTGTCCTGCTAATTTTTGTCTCGGGCAAGATTGTTCTGACTGGAGCGAAGGTGAGAGAACAGACGTATACTGCCTTCGAGAACATATATCC
CGTCCTCACAGAGTTCAGAAAAGTTCAGCAATGATGGTTGACTTTGGAAGTAAGCAGAATTGGAGATGTTGTTGCTTGCGGCC

Figure S11. TBPII sequence alignment and primer location

ADP-ribosylation factor (ADPR)

reference sequence: AB050957.1

forward primer: CTCATGGTTGGTCTCGATGC

reverse primer: ACATCCCAAACAGTGAAGCT

product size: 143 bp

contig number: CTG25287, percentage identity: ≥92%, E value: ≤1.03 x 10^{-22}

```
E value: 5.56 x 10-64, Identities: 97%, gaps 0/149

Query       1  ATGGGGCTCACGTTCACCAAGCTGTTCAGCCGCCTCTTCGCCAAGAAGGAGATGAGGATT  60
               |||||||||||||||  |||||||||||||||||||||||||||||||||||||||||||
Subject 21896  ATGGGGCTCACGTTCGGCAAGCTGTTCAGCCGCCTCTTCGCCAAGAAGGAGATGAGGATT  21955

Query      61  CTCATGGTTGGTCTCGATGCCGCTGGTAAGACCACCATCCTCTACAAGCTCAAGCTCGGA  120
               ||||||||||||||||||||||||||||||||||||||||||||||||||||||||||| 
Subject 21956  CTCATGGTTGGTCTCGATGCCGCTGGTAAGACCACCATCCTCTACAAGCTCAAGCTCGGC  22015

Query     121  GAGATCGTCACCACCATCCCAACCATCGG  149
               |||||||||||||||||||| ||||||||
Subject 22016  GAGATCGTCACCACCATCCCTACCATCGG  22044


E value: 1.03 x 10-22, Identities: 92%, gaps 0/78

Query     148  GGATTCAACGTCGAAACTGTTGAATACAAGAACATCAGCTTCACTGTTTGGGATGTGGGG  207
               ||||| |||||||| ||||| |||||||||||||||||||||||||||||||||||||||
Subject 22349  GGATTTAACGTCGAGACTGTCGAATACAAGAACATCAGCTTCACTGTTTGGGATGTGGGG  22408

Query     208  GGCCAAGACAAGATCAGG  225
               || ||||||||| | |||
Subject 22409  GGTCAAGACAAGGTAAGG  22426


E value 1.45 × 10-45      Identities: 94% Gaps: 0/123

Query     218  AGATCAGGCCCCTGTGGAGGCACTACTTCCAGAATACCCAAGGGCTCATTTTTGTTGTTG  277
               ||||||||||||| ||||||||||||||||||||||| || || ||||| ||||||||||
Subject 22602  AGATCAGGCCCCTTTGGAGGCACTACTTCCAGAATACACAGGGACTCATCTTTGTTGTTG  22661

Query     278  ACAGCAATGATAGGGAACGTGTTGTTGAGGCTAGAGATGAGCTCCACAGAATGCTGAATG  337
               |||||||||||||||| |||||||||||||||||||||||||||||||| ||||||||||
Subject 22662  ACAGCAATGATAGGGAGCGTGTTGTTGAGGCTAGAGATGAGCTCCACAGGATGCTGAATG  22721

Query     338  AGG  340
               |||
Subject 22722  AGG  22724

E value 1.89 × 10-50      Identities: 96% Gaps: 0/124

Query     338  AGGATGAGCTGCGTGATGCTGTGTTGCTTGTATTTGCAAACAAACAAGATCTTCCTAATG  397
               |||||||||| |||||||||||||||||||||||||||||||||||||||||||| ||||
Subject 22999  AGGATGAGCTACGTGATGCTGTGTTGCTTGTATTTGCAAACAAACAAGATCTTCCCAATG  23058

Query     398  CCATGAATGCTGCTGAAATCACCGACAAGCTTGGTCTGCACTCCCTGCGCCAGCGGCACT  457
               |||||||||| ||||||||||| |||||||||||||||||||||||||||||||||||||
Subject 23059  CCATGAATGCGGCTGAAATCACTGACAAGCTTGGTCTGCACTCCCTGCGCCAGCGGCACT  23118

Query     458  GGTA  461
               ||||
Subject 23119  GGTA  23122
```

```
oat sequence:
ATGGGGCTCACGTTCGGCAAGCTGTTCAGCCGCCTCTTCGCCAAGAAGGAGATGAGGATTCTCATGGTTGGTCTCGATGCCGCTGGTAAGACCACCATCCTCTACAA
GCTCAAGCTCGGCGAGATCGTCACCACCATCCCTACCATCGGATTTAACGTCGAGACTGTCGAATACAAGAACATCAGCTTCACTGTTTGGGATGTGGGGGGTCAAG
ACAAGATCAGGCCCCTTTGGAGGCACTACTTCCAGAATACACAGGGACTCATCTTTGTTGTTGACAGCAATGATAGGGAGCGTGTTGTTGAGGCTAGAGATGAGCTC
CACAGGATGCTGAATGAGGATGAGCTACGTGATGCTGTGTTGCTTGTATTTGCAAACAAACAAGATCTTCCCAATGCCATGAATGCGGCTGAAATCACTGACAAGCT
TGGTCTGCACTCCCTGCGCCAGCGGCACTGGTA
```

Figure S12. ADPR sequence alignment and primer location

Similar to phosphogluconate dehydrogenase (PGD)

reference sequence: Ta30797 (Unigene Cluster)

forward primer: GCAAAGATGAAACTGGTGGTCA
reverse primer: CAACCCACTTTTGTCCGCC

product size: 90 bp

contig number: CTG22048 percentage identity: ≥90%, E value: ≤1.58×10^{-28}

E value 1.06×10^{-30} Identities: 90% Gaps: 0/103

```
Query     217   GTTGTTTTCGACAAGGATGGCGTGCTCGAGCAAATCGGAGAGGGCAAGGGCTACGTGGAT  276
                |||||||||||||||||||||||||||||||||||||| || || |||||||| |||||
Subject 40058   GTTGTTTTCGACAAGGATGGCGTGCTCGAGCAAATCGGGGAAGGGAAGGGCTATGTGGAC  39999

Query     277   ATGTCCACTGTTGATGCTGCAACTTCTTGCAAGATAAGCGAGG  319
                |||||||| |||||||| || || |||||||||||||| ||||
Subject 39998   ATGTCCACCGTTGATGCCGCGACCTCTTGCAAGATAAGTGAGG  39956
```

E value 7.16×10^{-33} Identities: 91% Gaps: 0/105

```
Query     317   AGGCGGTTAAACAAAAGGGCGGAGCTTTTGTTGAAGCTCCAGTTTCAGGGAGCAAGAAGC  376
                |||| |||||||| || |  ||||||||||||||||||||||||||||||||||||||||
Subject 39013   AGGCAGTTAAACAGAAAGCTGGAGCTTTTGTTGAAGCTCCAGTTTCAGGGAGCAAGAAGC  38954

Query     377   CAGCTGAAGATGGCCAATTGGTCATTCTTGCTGCAGGCGACAAGG  421
                ||||||||||||| |||||||| |||||||| ||||| |||||||
Subject 38953   CAGCTGAAGATGGTCAATTGGTTATTCTTGCAGCAGGGGACAAGG  38909
```

E value 1.48×10^{-41} Identities: 93% Gaps: 0/118

```
Query     419   AGGCACTATATGATGATATGGTCCCTGCATTTGATGTACTTGGGAAGAAGTCATTCTTTC  478
                ||||||||||||| || ||||||||||| ||||||||||||||||| ||||| |||||
Subject 37645   AGGCACTATATGACGACATGGTCCCTGCCTTTGATGTACTTGGGAAAAAGTCGTTCTTCT  37586

Query     479   TGGGGGAGATGGGAAATGGAGCAAAGATGAAACTGGTGGTCAACATGATCATGGGAAG  536
                |||||||||| |||||||||||||||||||||||||||||||||||||||||||||||
Subject 37585   TGGGGGAGATTGGAAATGGAGCAAAGATGAAACTGGTGGTCAACATGATCATGGGAAG  37528
```

E value 1.58×10^{-28} Identities: 96% Gaps: 0/82

```
Query     535   AGTATGATGAATGCTTTTTCTGAGGGACTCTGTTTGGCTGACAAAAGTGGGTTGAGCCCC  594
                |||| ||||||||| ||||||||||||||||||||||| |||||||||||||||||||||
Subject 37031   AGTACGATGAATGCCTTTTCTGAGGGACTCTGTTTGGCGGACAAAAGTGGGTTGAGCCCC  36972

Query     595   CAGACGCTTCTTGATGTCCTGG  616
                ||||||||||||||||||||||
Subject 36971   CAGACGCTTCTTGATGTCCTGG  36950
```

```
oat sequence:
AGTGCGACGAGCTCGCCGCGCTGGGCGCTACCGTCGGGGACACGCCCGCGAAGGTCGTCGCCAAGTGCAACTACACCATCGCCATGCTCTCCGA
TCCCAGCGCCGCGCTATCCGTTGTTTTCGACAAGGATGGCGTGCTCGAGCAAATCGGGGAAGGGAAGGGCTATGTGGACATGTCCACCGTTGAT
GCCGCGACCTCTTGCAAGATAAGTGAGGCAGTTAAACAGAAAGCTGGAGCTTTTGTTGAAGCTCCAGTTTCAGGGAGCAAGAAGCCAGCTGAAG
ATGGTCAATTGGTTATTCTTGCAGCAGGGGACAAGGCACTATATGACGACATGGTCCCTGCCTTTGATGTACTTGGGAAAAAGTCGTTCTTCTT
GGGGGAGATTGGAAATGGAGCAAAGATGAAACTGGTGGTCAACATGATCATGGGAAGTACGATGAATGCCTTTTCTGAGGGACTCTGTTTGGCG
GACAAAAGTGGGTTGAGCCCCCAGACGCTTCTTGATGTCCTGG
```

Figure S13. PGD sequence alignment and primer location

Phenylalanine ammonia lyase (PAL)

reference sequence: MT150275.1

forward primer: GCAACTTCCAGGGCACCC

reverse primer: CTCCGAGAACTGAGCGAACAT

product size: 95 bp

```
sequence identity: 91%, E-value: 0.00, gaps: 0/1683, contig number: CTG14153
Query        1  CAGGTTCCTTAACGCCGGTGCCTTCGGAACTGGCACGGACGGACACGTTCTGCCCGCCGA  60
                ||||||||| |||||||||||||||||||| ||||||||||||||||| ||||| |||||
Subject 142817  CAGGTTCCTCAACGCCGGTGCCTTCGGAACCGGCACGGACGGACACGTCCTGCCGGCCGA  142758

Query       61  GGCAACACGCGCGGCTATGCTCGTCCGCATCAACACCCTCCTCCAGGGTTACTCGGGCAT  120
                |||||| ||||| || ||||| |||||||||||||||||||| |||||||||||||| ||
Subject 142757  GGCAACGCGCGCCGCCATGCTGGTCCGCATCAACACCCTCCTGCAGGGTTACTCGGGGAT  142698

Query      121  CCGCTTCGAGATCCTCGAGGCCATCACCAAGCTGCTCAACGCCAATGTCACGCCGTGCTT  180
                ||||||||||||||||||||||||||||||||||||||||||||| ||||| |||||| |
Subject 142697  CCGCTTCGAGATCCTCGAGGCCATCACCAAGCTGCTCAACGCCAACGTCACCCCGTGCCT  142638

Query      181  GCCGCTCCGGGGCACCATCACTGCATCCGGTGACCTTGTGCCGCTGTCCTACATTGCCGG  240
                 |||||||||||||||||||| || || || ||||| |||||||| ||||||||||| ||
Subject 142637  CCCGCTCCGGGGCACCATCACCGCCTCGGGCGACCTCGTGCCGCTCTCCTACATTGCGGG  142578

Query      241  CCTTATCACCGGCCGACAGAACTCTGTTGCGGTGGCCCCTGATGGCAGCAAGGTGACCGC  300
                |||||||||||| || |||||||| || || |||||||| ||||||| ||||||||||||
Subject 142577  CCTTATCACCGGTCGCCAGAACTCCGTCGCCGTGGCCCCCGATGGCACCAAGGTGACCGC  142518

Query      301  TGCCGAGGCATTCAAGATCGCCGGGATCGAGCACGGGTTCTTTGAGCTGCAGCCCAAGGA  360
                ||||||||| |||||||||||||| |||||||||||||||||||||||||||||||||||
Subject 142517  TGCCGAGGCGTTCAAGATCGCCGGCATCGAGCACGGGTTCTTTGAGCTGCAGCCCAAGGA  142458

Query      361  GGGCCTTGCCATGGTGAACGGCACGGCTGTGGGCTCTGGTCTTGCATCGACCGTGCTCTT  420
                 |||||||||||||||||||| || || ||||| || || ||||| ||||||||||| ||
Subject 142457  AGGCCTTGCCATGGTGAACGGGACAGCCGTGGGGTCGGGACTTGCGTCGACCGTGCTGTT  142398

Query      421  TGAGGCCAATGTTCTGTCCGTCCTTGCTGAGGTCTTGTCTGCGGTGTTCTGCGAGGTGAT  480
                |||||||||  | ||  || | || ||||||||| |||| ||||| ||||||||||||||
Subject 142397  TGAGGCCAACATCCTCGCCATACTCGCTGAGGTCCTGTCCGCGGTCTTCTGCGAGGTGAT  142338

Query      481  GAACGGCAAGCCGGAGTTCACTGACCACCTGACCCACAAGCTGAAGCACCACCCTGGACA  540
                || |||||||||||||||||| ||||||||||||||||||||||||||||||||||| ||
Subject 142337  GACCGGCAAGCCGGAGTTCACGGACCACCTGACCCACAAGCTGAAGCACCACCCTGGGCA  142278

Query      541  GATTGAAGCAGCTGCAATCATGGAGCACATCTTGGAGGGAAGCTCATACATGAAGCAGGC  600
                ||| || || || || |||||||||||||||||||| || ||||| ||||||||||||||
Subject 142277  GATCGAGGCGGCGGCGATCATGGAGCACATCTTGGAAGGGAGCTCGTACATGAAGCAGGC  142218

Query      601  AAAGAAGCTTGGCGAGCTCGACCCCTTGATGAAGCCGAAGCAGGACCGGTATGCCCTCCG  660
                 ||||  |  ||||||||||||||  ||||||||||||||||||||||||| || |||||
Subject 142217  CAAGATACAGGGCGAGCTCGACCCGCTGATGAAGCCGAAGCAGGACCGGTACGCGCTCCG  142158

Query      661  CACTTCGCCTCAGTGGCTCGGCCCACAAATCGAAGTTATTCGATTTGCCACCAAGTCGAT  720
                ||| ||||| |||||||| ||||| || ||||| ||||| || || ||||||||||||||
Subject 142157  CACGTCGCCGCAGTGGCTTGGCCCTCAGATCGAGGTTATCCGCTTCGCCACCAAGTCGAT  142098

Query      721  TGAGCGTGAGATCAACTCTGTCAACGACAACCCGCTCATCGACGTCTCCCGTGGCAAGGC  780
                 ||||| ||||||||||| ||||||||||||||||||||||| |||||||||||||||||
Subject 142097  CGAGCGCGAGATCAACTCCGTCAACGACAACCCGCTCATCGATGTCTCCCGTGGCAAGGC  142038

Query      781  CATCCACGGTGGCAACTTCCAAGGCACCCCCATTGGTGTCTCCATGGACAACACCCGCCT  840
                 ||||| || ||||||||||| ||||||||||| ||||||||||||||||||||||||||
Subject 142037  GATCCATGGGGGCAACTTCCAGGGCACCCCCATCGGTGTCTCCATGGACAACACCCGCCT  141978

Query      841  CGCCATTGCTGCTATTGGCAAGCTGATGTTCGCGCAGTTCTCGGAGCTCGTGAACGACTT  900
                 ||||| ||||||||||||||||| ||||||||||||||||||||||| |||||||||||
Subject 141977  TGCCATCGCTGCTATTGGCAAGCTCATGTTCGCGCAGTTCTCGGAGCTGGTGAACGACTT  141918

Query      901  CTACAACAATGGCCTGCCTTCCAACCTGTCCGGTGGGCGCAACCCGAGCTTGGACTATGG  960
                ||||||||| ||||| || |||||||||||||||||||||||||||||||||||||| ||
Subject 141917  CTACAACAACGGCCTCCCGTCCAACCTGTCCGGTGGGCGCAACCCGAGCTTGGACTACGG  141858
```

```
oat sequence:
CAGGTTCCTCAACGCCGGTGCCTTCGGAACCGGCACGGACGGACACGTCCTGCCGGCCGAGGCAACGCGCGCCGCCATGCTGGTCCGCATCAACACCCTCCTGCAGG
GTTACTCGGGGATCCGCTTCGAGATCCTCGAGGCCATCACCAAGCTGCTCAACGCCAACGTCACCCCGTGCCTCCCGCTCCGGGGCACCATCACCGCCTCGGGCGAC
CTCGTGCCGCTCTCCTACATTGCGGGCCTTATCACCGGTCGCCAGAACTCCGTCGCCGTGGCCCCCGATGGCACCAAGGTGACCGCTGCCGAGGCGTTCAAGATCGC
CGGCATCGAGCACGGGTTCTTTGAGCTGCAGCCCAAGGAAGGCCTTGCCATGGTGAACGGGACAGCCGTGGGGTCGGGACTTGCGTCGACCGTGCTGTTTGAGGCCA
ACATCCTCGCCATACTCGCTGAGGTCCTGTCCGCGGTCTTCTGCGAGGTGATGACCGGCAAGCCGGAGTTCACGGACCACCTGACCCACAAGCTGAAGCACCACCCT
GGGCAGATCGAGGCGGCGGCGATCATGGAGCACATCTTGGAAGGGAGCTCGTACATGAAGCAGGCCAAGATACAGGGCGAGCTCGACCCGCTGATGAAGCCGAAGCA
GGACCGGTACGCGCTCCGCACGTCGCCGCAGTGGCTTGGCCCTCAGATCGAGGTTATCCGCTTCGCCACCAAGTCGATCGAGCGCGAGATCAACTCCGTCAACGACA
ACCCGCTCATCGATGTCTCCCGTGGCAAGGCGATCCATGGGGGCAACTTCCAGGGCACCCCCATCGGTGTCTCCATGGACAACACCCGCCTTGCCATCGCTGCTATT
GGCAAGCTCATGTTCGCGCAGTTCTCGGAGCTGGTGAACGACTTCTACAACAACGGCCTCCCGTCCAACCTGTCCGGTGGGCGCAACCCGAGCTTGGACTACGG
```

Figure S14. PAL sequence alignment and primer location
