# Supplementary material for: Validation of Reference Genes for Studying Different Abiotic Stresses in Oat (Avena sativa L.) by RT-qPCR
Source: Plants (Basel). 2021 Jun 22;10(7):1272. doi: 10.3390/plants10071272 (PMC8309034; doi:10.3390/plants10071272)
Supplement: Supplementary file 1 [file plants-10-01272-s001.zip › Table S1, sequence homology.pdf]

Table S1. Sequence homology between wheat (NCBI GeneBank) and the corresponding oat (*Avena sativa* L.) sequences (Bioproject 541449)

Gene Abbreviation	Accession No. (NCBI)	Percentage of Indentity	E value	Contig number (BioProject 541449)
ACT	MF405765.1	95%	0.00	CTG107765
TUB	U76558.1	93%	0.00	CTG25086
CYP	EU035525.1	92%	0.00	CTG81232
GAPDH	KR029492.1	≥87%	$\leq 6.38*10^{-18}$	CTG108160
UBC	M62720.1	≥93%	$\leq 1.74 \text{ x } 10^{-30}$	CTG29477
EF1	M90077.2	92%	0.00	CTG31755
TBP	L07604.1	≥90%	$\leq 5.56*10^{-07}$	CTG9379
ADPR	Ta2291 (Unigene cluster)	≥92%	$1.03 \text{ x } 10^{-22}$	CTG25287
PGD	Ta30797 (Unigene cluster)	≥90%	$\leq 1.58 \times 10^{-28}$	CTG22048
PAL	MT150275.1	91%	0.00	CTG14153
